# Strain-Dependent Adhesion Variations of *Shouchella clausii* Isolated from Healthy Human Volunteers: A Study on Cell Surface Properties and Potential Probiotic Benefits

**DOI:** 10.3390/microorganisms12091771

**Published:** 2024-08-27

**Authors:** Tanisha Dhakephalkar, Vaidehi Pisu, Prajakta Margale, Siddhi Chandras, Deepa Shetty, Shilpa Wagh, Sumit Singh Dagar, Neelam Kapse, Prashant K. Dhakephalkar

**Affiliations:** 1 Hi Tech BioSciences India Ltd., Research & Development Centre, Plot No. 6 and 8, Ambadvet Industrial Estate, PO Paud, Pune 412108, Maharashtra, India; 2 Bioenergy Group, MACS-Agharkar Research Institute, G.G. Agarkar Road, Pune 411004, Maharashtra, India; 3 Department of Microbiology, Savitribai Phule Pune University, Ganeshkhind Rd., Aundh, Pune 411007, Maharashtra, India

**Keywords:** *Shouchella clausii*, adhesion, hydrophobicity, aggregation, pathogen exclusion, genome

## Abstract

The probiotic potential of *Shouchella clausii* is widely recognized, but little is known about its adhesive properties. Hence, this study aims to investigate the adhesion potential and cell surface properties of four human-origin *S. clausii* strains (B619/R, B603/Nb, B106, and B637/Nm). We evaluated epithelial adhesion, Extracellular Matrix (ECM) binding, aggregation ability, and cell surface hydrophobicity and used genome analysis for validation. Our results demonstrate that adhesion capability is a strain-specific attribute, with significant variations observed among the four strains. B619/R, B603/Nb, and B106 displayed stronger adhesion properties than B637/Nm. Supplementary adhesion assays showed that B637/Nm displayed high hydrophobicity, significant auto-aggregation, and significant mucin-binding abilities. Conversely, B619/R, B603/Nb, and B106 had mildly hydrophobic surfaces and low aggregation abilities. Genome annotation revealed the presence of various adhesion proteins in four strains. Notably, the reduced adhesion potential of B637/Nm was supported by the absence of the cell wall surface anchor family protein (LPxTG motif), which is crucial for interactions with intestinal epithelial cells or mucus components. Further, docking studies provided insights into the interaction of adhesion proteins with gut mucins. These findings contribute to a better understanding of how *S. clausii* strains interact with the gut environment, facilitating the development of probiotic formulations tailored for improved gut health and well-being.

## 1. Introduction

Probiotics are live microorganisms that, when administered in sufficient quantities, provide health advantages to the host [1]. These microorganisms are commonly employed to promote gastrointestinal health, enhance immune function, treat specific diseases, etc. For a strain to qualify as a probiotic, it must possess key attributes, such as acid and bile tolerance, survival through the gastrointestinal tract, the ability to adhere to intestinal epithelial cells, antimicrobial efficacy against potentially pathogenic bacteria, and favorable technological properties. The strains should also be safe for the intended use. Finally, it is crucial for probiotic strains to maintain viability at an effective dosage throughout the product’s shelf life [2]. This ensures that the strains remain viable and active when consumed. The most commonly used probiotics include members of non-sporulating genera, such as lactobacilli and *Bifidobacterium*, and yeast, *Saccharomyces* [3]. However, non-sporulating probiotics have technological challenges, particularly related to their survival and viability during oral administration and storage. Chemical and physical obstacles encountered during oral administration present a challenge for probiotic delivery technologies [4]. Chemical challenges encompass the acidity of the stomach and the presence of bile salts, which can inactivate the probiotic bacteria. Physical challenges include the rapid transit through the gastrointestinal tract (GIT), which limits probiotic retention in the intestines and hinders their adhesion and growth. These challenges may reduce the efficacy of non-sporulating probiotics in exerting their health benefits. Sporulating probiotics, such as *Bacillus* and *Clostridium* species, adopt spore formation as a survival tactic to tolerate harsh environmental conditions [5]. These spores are highly resilient and can maintain viability during distribution and storage. The ability to form spores allows these probiotic bacteria to survive the acidic pH of the GIT and reach the small intestine unharmed.

*Bacillus* species are viable alternatives to Lactic Acid Bacteria (LAB) for use as probiotics [6], considering the information presented above. *B. subtilis*, *B. polyfermenticus*, *B. clausii*, some *B. cereus*, *B. coagulans*, *B. pumilus*, and *B. licheniformis* species make up most commercial *Bacillus* probiotics, such as Bio-Kult^®^, Lactipan Plus, Bibactyl, Biosubtyl, Bispan^®^, Domuvar^®^, Enterogermina^®^, Lactopure, Lactospore, Neolactoflorene^®^, Sustenex^®^, Bactisubtil^®^, etc. In India, several spore-forming *Bacillus clausii* probiotics are available OTC (over the counter) for human use, such as ECOGRO^®^, ENTEROGERMINA^®^, ENTROMAX^®^, OSPOR^®^, GUTPRO^®^, CYFOLAC^®^, BACIPRO^®^, β-LOCK^®^, BENEGUT^®^, PROCILLUS^®^, PROALANA-B^®^, and TUFPRO^®^. It is commonly used as a medical supplement for treating acute diarrhea in adults and children, as well as adjunctive therapy for *Helicobacter pylori* infection in India.

*Bacillus clausii*, a spore-forming probiotic previously reclassified as *Alkalihalobacillus clausii* and now reclassified as *Shouchella clausii* [7], offers several technological advantages over non-sporulating probiotics. Its ability to form spores enhances its survival during food processing and GIT transit [8]. The spore-forming ability of *S. clausii* also contributes to its resistance to commonly used antibiotics [9]. Additionally, *S. clausii* has also demonstrated antimicrobial activity against pathogens such as *Clostridium difficile,* mucosal barrier-enhancing properties and immunomodulatory potential [10]. Its effectiveness in treating various conditions has been explored in numerous studies. A systematic review and meta-analysis of randomized controlled trials found that *S. clausii* significantly reduced the duration of acute childhood diarrhea and hospitalization compared to the control groups [11]. Another study demonstrated the cytoprotective effects of *S. clausii* against oxidative stress in cell culture and *in vivo* models [12]. These findings highlight the potential of *S. clausii* as an effective probiotic for various health conditions. The synergistic effect of *S. clausii* with prebiotics further expands its potential applications. These technological advantages make *S. clausii* a promising probiotic candidate for various therapeutic and functional food purposes.

Numerous *in vitro* and preclinical studies have underscored the probiotic potential of *S. clausii*, focusing on their ability to survive harsh gastric conditions and display health-promoting and disease-preventing traits, etc. Despite these studies, there remains a scarcity of research documenting the adhesion potential of *S. clausii*. The ability of probiotics to adhere to the gut epithelium is a crucial determinant of their effectiveness. Successful adhesion to intestinal epithelial cells is pivotal for establishing colonization and fostering interactions with host cells. Therefore, it is necessary to conduct a comprehensive evaluation of these traits. Many models have been devised to evaluate the adhesive potential of probiotics. These include binding to intestinal cell lines [13], intestinal mucus [14], extracellular matrix (ECM) proteins, and resected colonic tissue [15]. Although none of these models accurately capture the intricate interactions that take place in the mucosal layer of the digestive tract, they do provide a quick way to screen strains, and in most cases, there is a strong correlation between them. Ahire et al. [16] investigated the adherence of both vegetative *S. clausii* UBBC07 cells and spores to solvents and porcine mucin. Meanwhile, Jeon et al. [17] observed that *S. clausii* ATCC 700160 exhibits auto-aggregation and co-aggregation with pathogens. Mazzantini et al. [18] evaluated the adherence of four strains of *S. clausii* from Enterogermina to mucin. However, there has been no detailed study documenting the surface properties and adhesion ability of *S. clausii* to the components of the extracellular matrix and intestinal cell lines.

Strain-specific variations in adhesion behavior have been observed in various probiotic species [19], highlighting the importance of studying each strain comprehensively to evaluate its probiotic potential. Thus, to obtain insight into the possible adhesion mechanism, this study evaluated the adhesion potential of four strains of *S. clausii in vitro* and at the genome level.

## 2. Materials and Methods

### 2.1. Bacterial Strains and Culture Conditions

The BC4 consortium, comprising four strains of *Shouchella clausii*, was obtained from Hi Tech BioSciences India Pvt. Ltd., located in Pune, India. The consortium consists of four isolates: B619/R, B603/Nb, B106, and B637/Nm, which have been deposited in the National Center for Microbial Resource (NCMR), Pune, India, under the accession numbers MCC 0190, MCC 0189, MCC 0191, and MCC 0188, respectively. These strains were derived from healthy human volunteers. All the *S. clausii* strains were cultured in Brain Heart Infusion (BHI) at 37 °C for 24 h.

### 2.2. Characterization of S. clausii Strains

The effect of temperature (22, 30, 37, 45, and 60 °C), pH (2, 3, 5, 7, 8, and 10), and salinity (2% to 10%) on the growth of the *S. clausii* strains was evaluated by inoculating 10^8^ cells/mL of each of the strains in Brain Heart Infusion (BHI) medium followed by incubation for 24–48 h at varying temperatures and at 37 °C in case of the pH and salinity experiments. Growth was recorded by measuring the OD of the strains after 24 h at 600 nm. The ability of *S. clausii* strains to survive harsh gastrointestinal conditions was investigated following a method similar to that described by Vecchione et al. [20] with a few modifications. Each of the four strains was independently evaluated for its tolerance to simulated gastric fluid (SGF) and simulated intestinal fluid (SIF). Vegetative cells as well as spores of each of the strains (10^10^/mL) were exposed independently and individually to SGF (0.03 M sodium chloride, 0.084 M hydrochloric acid, and 0.32% *w*/*v* pepsin at pH 2.5) for 0, 30, 60, and 120 min and to SIF (0.3% *w*/*v* ox gall bile salts, 0.1% *w*/*v* pancreatin in sterile 0.85% saline solution at pH 8) for 0, 120, 240, and 360 min. Viable cell counts were determined at each time point using the spread plate method on BHI medium supplemented with 2% (*w*/*v*) agar [16].

The antimicrobial activity of *S. clausii* strains against the pathogens, namely *Staphylococcus aureus* subsp. *aureus* ATCC 6538, *Enterococcus faecalis* ATCC 29212, *Escherichia coli* ATCC 8739, *Salmonella* Typhimurium ATCC 13311, *Bacillus subtilis* ATCC 6051, and *Pseudomonas aeruginosa* ATCC 10145, was evaluated by agar spot testing according to Jacobsen et al. [21]. Briefly, 5 μL of each of the *S. clausii* strains (10^8^ cells/mL) was spot inoculated on BHI medium supplemented with 2% (*w*/*v*) agar and incubated at 37 °C for 24 h to allow colonies to develop. Furthermore, the overnight-grown pathogenic cultures with a cell count of 10^8^ cells/mL were mixed with 9 mL soft BHI medium supplemented with 0.7% (*w*/*v*) agar and overlaid on an agar plate with colonies of *S. clausii* strains individually and incubated for (24 h–48 h). The zone of inhibition (in mm) was recorded as antimicrobial activity. The bile salt hydrolase activity was determined as described by Ahire et al. [16]. The proteolytic and lipolytic activities of the strains were tested as previously reported by Lim et al. and Vijayakumari et al., respectively [22,23]. *S. clausii* strains were spotted on skimmed milk agar and tributyrin agar, respectively. Both plates were incubated at 37 °C. A clear zone around the colony on agar plates indicated enzyme activity. Beta-galactosidase activity was evaluated using CLED (Cystine Lactose Electrolyte Deficient) agar. *S. clausii* strains were streaked onto the agar plate and incubated at 37 °C for 24–48 h, with colonies exhibiting enzyme activity producing a yellow color due to lactose hydrolysis-induced pH change.

The antioxidant potential of the strains was investigated by their 1,1-diphenyl-2-picrylhydrazyl (DPPH) free radical scavenging activity, as described by Mu et al. [24]. Intact cells of *S. clausii* were harvested, washed, and suspended in PBS to attain a concentration of 10^9^ cells/mL. The DPPH assay involved mixing 1.0 mL of bacterial suspension with 1.0 mL of a 0.2 mM DPPH-ethanol solution, followed by incubation at 25 °C in dark for 30 min. Absorbance at 517 nm was measured, and the DPPH scavenging activity (%) was calculated. Ascorbic acid (10 μg) served as the positive control.

The cholesterol assimilation ability was determined as per the methods given by Tarrah et al. [25]. *S. clausii* strains were incubated in BHI prepared in SIF and supplemented with water-soluble cholesterol for 24 h. The residual cholesterol was measured, and the percentage of cholesterol assimilated was calculated to assess anti-hypercholesterolemic activity. Exopolysaccharide production was tested according to the method previously described by Midik et al. [26]. The antibiotic susceptibility/resistance of *S. clausii* strains was evaluated using E-test strips. In brief, 100 μL of each *S. clausii* suspension (10^8^ cells/mL) was spread onto BHI agar. Each antibiotic E-test strip was placed at the center of an agar plate and incubated at 37 °C for 24 h. Susceptibility or resistance to each antibiotic was determined based on CLSI microbiological cut-off values.

### 2.3. Adhesion of S. clausii Strains to HT-29 and Caco-2 Cell Lines

#### 2.3.1. Cell Adhesion Assay

The adhesion of *S. clausii* strains to HT-29 and Caco-2 cell lines was carried out as per the methods described by Sharma and Kanwar and Lebeer et al. [27,28], respectively, with a few modifications. For the adhesion assay, 1 mL of the *S. clausii* strain (1 × 10^8^ CFU/mL) was added onto the coverslips placed in tissue culture plates having a monolayer of HT-29 cells/Caco-2 cells and incubated at 37 °C for 2 h (HT-29) and 1.5 h (Caco-2) in a CO_2_ chamber. After incubation, each well was gently washed twice with PBS, fixed, and observed using scanning electron microscopy [29]. The cell lines used in this study were purchased from NCCS (Pune, India) and maintained in Dulbecco’s modified eagle medium (DMEM, Gibco: 11965092, Waltham, MA, USA) supplemented with 9% fetal bovine serum and 1% antibiotic and antimycotic solution in a CO_2_ incubator at 37 °C.

#### 2.3.2. Competitive Exclusion of *Salmonella* Typhimurium Adhesion from HT-29 Cell Line Using the *S. clausii* Strains

The exclusion assay was performed as per Choi et al. [30], with some modifications. Briefly, the HT-29 cells were first incubated with *S. clausii* suspension (10^8^ cells/mL) for 1 h at 37 °C in a 5% CO_2_ atmosphere. After incubation, the plate was removed, and the media were discarded and washed with PBS twice with gentle agitation. Subsequently, *Salmonella* Typhimurium (10^8^ cells/mL) was added to each well and incubated for 1 h at 37 °C in a 5% CO_2_ atmosphere. After incubation, each well was gently washed twice with PBS, then treated with methanol and kept at room temperature for 10 min. Methanol was removed, and the wells with the coverslips were allowed to air dry for 2 min. The coverslips were then subjected to Gram staining. Cells were observed under a 100X objective lens of a light microscope. Then, 25 images of random fields were captured to count the adhered number of bacteria per HT-29 cell. The final results were expressed as the number of bacterial cells adhered per 100 HT-29 cells. The adhesion of pathogenic bacteria alone to HT-29 cells was assigned to 100% (control), and changes in the adhesion of pathogenic bacteria when treated with *S. clausii* cultures were determined.

#### 2.3.3. Adhesion to ECM Components

The *S. clausii* strains were tested for binding to different substrates (mucin, fibrinogen, and collagen) immobilized on 96-well plates using the method described by Muñoz-Provencio et al. [31] with a few modifications. Mucin (500 µg/mL), fibrinogen (50 µg/mL), and collagen (50 µg/mL) were used as substrates. The microtiter plates were coated with 200 µL of each of the three substrates and incubated overnight at 4 °C. After immobilization, the wells were washed three times with PBS and blocked with BSA for 2 h at room temperature. After incubation, the wells were washed with PBS once. The OD600 nm of overnight-grown *S. clausii* strains was adjusted to 1 with PBS. 200 µL of each of the strains was added to each well, followed by incubation at 37 °C for 2 h. The non-adhered cells were removed by washing three times with 200 µL of PBS + 0.05% Tween 20, and the plates were dried at 55 °C. The adhered cells were stained with crystal violet 1 mg/mL (200 µL/well) for 45 min. The colorant was released using 50 mM citrate buffer pH 4.0 (200 µL/well) for 45 min after six PBS washes, and the absorbance was measured at 595 nm. The control wells were without the *S. clausii* strains. Adhesion was expressed as the optical density (OD595 nm) of stained cells.

#### 2.3.4. Determination of Cell Surface Characteristics

##### Cell Surface Hydrophobicity

To determine the surface hydrophobicity of *S. clausii* strains, the bacterial adherence to hydrocarbons (BATH) assay described by Rokana et al. [32] was employed. Briefly, 3 mL of *S. clausii* suspension (initial ODi ~1) was mixed with 1 mL of various hydrocarbons (chloroform, toluene, ethyl acetate, xylene, n-hexane, and hexadecane) and incubated at 37 °C for 10 min. After vortexing for 15 s, the mixture was allowed to settle undisturbed at 37 °C for 30 min to facilitate phase separation. The aqueous phase was carefully collected, and OD at 600 nm (ODt) was measured. The hydrophobicity percentage, indicating cell adherence to hydrocarbons, was calculated using the formula:

% Hydrophobicity = (ODi − ODt/ODi) × 100.

##### Auto-Aggregation and Co-Aggregation Assay

The auto-aggregation assay, following Han et al. [33], involved dispensing 1 mL of *S. clausii* cell suspension (initial OD600 ≈ 1.0) (A0) into tubes, which were then incubated at 37 °C without agitation. Absorbance readings at 600 nm were taken hourly for 4 h by withdrawing settled supernatant (At). For the co-aggregation assay, as per Collado et al. [34], cell suspensions of *S. clausii* and various pathogens were prepared with an OD600 of 1.0. Equal volumes of *S. clausii* and pathogen suspensions of each of *E. coli*, *S. aureus* subsp. *aureus*, *E. faecalis*, *S. dysenteriae*, *K. pneumoniae*, *P. aeruginosa*, *E. aerogenes*, and *S.* Typhimurium were mixed individually and incubated at 37 °C. Absorbance at 600 nm was measured at 1-h and 4-h intervals. Percentages of auto-aggregation and co-aggregation were calculated using the formulas:

% Auto-aggregation = (1 − At/A0) × 100

% Co-aggregation = [(OD_Path_ + OD_Cul_) − OD_Mix_/(OD_Path_ + OD_Cul_)] × 100

### 2.4. Genome Sequencing and Annotation

*S. clausii* strains B619/R, B106, B603/Nb, and B637/Nm genomes were sequenced on Genestudio Ion S5 Plus following the manufacturer’s guidelines. *De novo* assembly for all four strains was performed using the Spades assembler (version 3.10) with default settings. Functional annotation was conducted using the Rapid Annotations using Subsystems Technology (RAST) server [35]. Genes associated with adhesion potential were identified and extracted from the annotated data. The PlasmidFinder 2.1 database was used to identify plasmids in the genomes of strains B619/R, B106, B603/Nb, and B637/Nm [36]. The virulence genes in the genomes of *S. clausii* strains were detected using the webtool Virulencefinder v2.0 (https://cge.food.dtu.dk/services/VirulenceFinder/ (accessed on 6 June 2023)) [37]. BAGEL 4.0 software was used to predict bacteriocins in the genomes of *S. clausii* strains [38]. The whole-genome shotgun project of *S. clausii* B619/R, B603/Nb, B106, and B637/Nm has been deposited at DDBJ/ENA/GenBank under the accession numbers JABFCW000000000, JABFCU000000000, NFZO00000000, and JABFCV000000000, respectively. The versions described in this paper are JABFCW020000000, JABFCU020000000, NFZO02000000, and JABFCV020000000, respectively.

### 2.5. In Silico Characterization of Cell Surface Protein

The molecular weight, theoretical isoelectric point (pI), number of positive and negative residues (+R/−R), aliphatic index (AI), extinction coefficient (ε), instability index (II), and grand average hydropathy (GRAVY) were calculated using Expasy’s Protparam server (https://web.expasy.org/protparam/ (accessed on 10 January 2023)). I-TASSER was used for homology modeling of the protein’s three-dimensional structure [39].

### 2.6. Molecular Docking Studies

Molecular docking was implemented to evaluate the interaction and protein-ligand-binding affinity between the key adhesion molecule of *S. clausii* and intestinal mucins. The mucus layer comprises various mucins, including MUC1, MUC3, MUC4, MUC12, and MUC13. The three-dimensional structures of mucins were obtained from the Swiss Model Database [40]. To examine the interaction between the cell wall anchor family protein (LPxTG) and MUC1, MUC3, MUC4, MUC12, and MUC13, the ClusPro protein-protein docking web server [41] was employed. The docking process involved matching the proteins and recording their scores, which were represented as lower energy values expressed in kilocalories per mole (kcal/mol). Subsequently, the most favorable affinity score for each ligand-protein interaction was selected.

### 2.7. Statistical Analysis

The experiments were performed in triplicates and the results are expressed as mean ± standard deviation (SD).

## 3. Results and Discussion

Studies have shown that the effectiveness and functionality of probiotics vary depending on the strain [42]. Thus, this study aimed to explore the probiotic potential of multiple strains of *Shouchella clausii* (B619/R, B603/Nb, B106, and B637/Nm), focusing predominantly on their adhesive abilities. These four strains were derived from healthy human volunteers and were used to prepare a probiotic formulation by the company.

Each of the four Gram-positive, aerobic, sporulating strains used in the present study exhibited a high degree of SSU rRNA gene sequence homology (>99%) with the closest phylogenetic affiliate, *S. clausii* DSM 8716. The identity of each strain was also confirmed by their genome sequence analysis, which revealed ~95.4% ANI and ~62.5% DDH homologies with the reference genome of DSM 8716, revealing a marked difference between the strains. The strains were able to grow well at a temperature, pH, and salinity range of 22–45 °C, 7.0–10.0, and 2–10%, respectively.

### 3.1. In Vitro Resistance of S. clausii Strains to Simulated Gastric and Intestinal Fluids

In order to colonize the gut and exert their beneficial effects, it is crucial for the probiotic strains to tolerate gastrointestinal stress, which includes exposure to gastric acidity, digestive enzymes, and bile salts [43]. Probiotics that can withstand the harsh conditions in the stomach have a higher likelihood of reaching the intestines in viable and active forms, where they can exert their beneficial effects [44]. Hence, in the present study, vegetative cells as well as spores of *S. clausii* strains were exposed to SGF and SIF (and not to individual enzymes of acidic pH) individually and separately to determine their potential to survive gastric transit. The spore suspensions of all four strains displayed the ability to survive the SGF (pH 1.4, 0.32% (*w*/*v*) pepsin, up to 120 min) and intestinal conditions (0.3% (*w*/*v*) Ox-Bile, 0.1% (*w*/*v*) pancreatin, pH 8.0, up to 360 min) without experiencing a significant reduction in viable count after exposure (Table 1). A declining trendline was noticed for the total counts of each strain after 30-, 60-, and 120-min incubations in the SGF. Even after 120 min of exposure to SGF, all test strains maintained survival above 55%, with strain B106 exhibiting the highest survival at 95%. Similar results were obtained on exposure to artificial simulated intestinal fluid (SIF) containing the pancreatin-bile salt solution (pH 8.0). At the end of the incubation in the SIF, 71, 36, 66, and 71% survival rates were registered for B619/R, B603/Nb, B106, and B637/Nm, respectively (Table 1). Overall, the harsh GIT tolerance of the isolates in this study was found to be strain-specific. On the other hand, the vegetative cells of the same strains were found to be sensitive to the harsh conditions of SGF and SIF. This contrast highlighted the superiority of sporulating cultures over non-sporulating ones in terms of their ability to better tolerate gastric stress. These findings aligned with previous reports on the survival and persistence of *S. clausii* in the human gastrointestinal tract following oral administration as a spore-based probiotic formulation. Ahire et al. [16] demonstrated the high resistance of *S. clausii* spores to acidic conditions as compared to vegetative cells, wherein vegetative cells displayed reduced survivability within an hour to pH 1 and 2 [16]. Another study by Cenci et al. [45] showed that *S. clausii* spores could tolerate pH 2, while vegetative cells could tolerate pH ≤ 4. Khokhlova et al. [12] reported the tolerance of *S. clausii* strains to SGF. However, in that study, the pH of SGF was 3.0, which may not truly represent gastric acidity [12]. In the present study, the pH of SGF was adjusted to 1.4 to truly represent the gastric fluid. The survival of the *S. clausii* strains used in the present study truly represented their ability to survive gastric transit. Thus, the ability of the four *S. clausii* strains to tolerate GIT stress underscored their potential to colonize and effectively function in the gut environment.

### 3.2. Safety Assessment of S. clausii Strains

In addition to surviving gastric transit, the susceptibility of probiotics to antibiotics is also an essential consideration. Antibiotic resistance is a growing concern, and alternative treatments for infections, such as *Helicobacter pylori*, are being explored [46]. Considering safety aspects, the presence of transferrable antibiotic resistance genes should be given consideration, as recommended by the Qualified Presumption of Safety (QPS) approach endorsed by the European Food Safety Authority (EFSA). Susceptibility to different antibiotics was assayed by the microbroth dilution method, and strain-specific sensitivity phenotypes were observed in the present study. B619/R was found to be resistant to Neomycin, whereas the other strains were sensitive. While the other strains were susceptible to rifampicin, B603/Nb was found to be resistant. Chloramphenicol resistance was observed in all four strains. The highest MIC value was found for B106; however, the MIC values were significantly diverse, indicating that the levels of resistance to chloramphenicol varied amongst the four strains. B637/Nm was resistant to tetracycline, whereas other strains were susceptible. All the *S. clausii* strains were resistant to Clarithromycin and Metronidazole, the antibiotics administered to treat *H. pylori* infection (Table 1). However, the observed resistance to multiple antibiotics in B619/R, B603/Nb, B106, and B637/Nm was found to be intrinsic in nature and consistent with the antibiotic resistance profile reported earlier for Enterogermina strains by Abbrescia et al. [47]. The intrinsic resistance of *S. clausii* to clindamycin, erythromycin, and chloramphenicol was also demonstrated by Lakshmi et al. [48]. Khatri et al. also reported the presence of genes conferring resistance to chloramphenicol, streptomycin, rifampicin, and tetracycline in the *S. clausii* ENTPro strain [49].

On the other hand, the four strains exhibited significant sensitivity to a range of antibiotics. This included extended spectrum Penicillin (Piperacillin, Ampicillin, Amoxicillin), Carbapenems (Imipenem, Meropenem), second- and third-generation tetracyclines (Doxycycline, Minocycline, Tigecycline), Aminoglycosides (Amikacin, Gentamicin, Netilmicin), and Ciprofloxacin and Trimethoprim. Strains B603/Nb, B106, and B637/Nm displayed sensitivity to Tobramycin, while B619/R was found to be resistant. In addition to this, B637/Nm was also found to be sensitive to Levofloxacin and second- and third-generation Cephalosporins (Cefuroxime and Ceftriaxone). While antibiotic resistance alone may not pose a safety concern, the possibility of resistance transfer is problematic. To address this, the study confirmed the absence of plasmids in the genomes of B619/R, B603/Nb, B106, and B637/Nm using Plasmid Finder (v2.0.1), eliminating the potential for the horizontal transfer of antibiotic resistance genes to other pathogens. The multiple antibiotic resistance of *S. clausii* strains makes them suitable for co-administration with antibiotics to restore a healthy gut microbiota. Such an oral probiotic supplement can prevent antibiotic-induced diarrhea in patients under antibiotic treatment [47]. The genomes of *S. clausii* strains were mined for virulence factors belonging to *S. aureus*, *Enterococcus*, *Listeria*, and *Escherichia coli* using VirulenceFinder 2.0. Genes or their variants encoding the virulence factors were also absent in the genomes of all four strains, further confirming the GRAS nature of the *S. clausii* strains.

### 3.3. Disease-Preventing Traits of S. clausii Strains

In terms of disease-preventing characteristics, the strains displayed antimicrobial activity against enteric pathogens. Among the quartet of strains, each exhibited antimicrobial efficacy against *P. aeruginosa*. Notably, B603/Nb showcased a more robust inhibition zone of 27 mm against *P. aeruginosa* compared to the 14.5 ± 0.7 mm of B619/R, 11 ± 0.8 mm of B106, and 15.6 ± 1.1 mm of B637/Nm. B106, B603/Nb, and B637/Nm displayed activity against *S. aureus* subsp. *aureus*. Furthermore, B106 and B637/Nm displayed effectiveness against *E. faecalis* (Table 1). Urdaci et al. reported anti-staphylococcal activity but not anti-*Salmonella* activity of *S. clausii* strains [50]. Our results agree with this study. Sharma et al. documented the antimicrobial activity of *S. clausii* against different pathogens, such as *E. coli*, *S. aureus*, and *P. aeruginosa* [51]. In the present investigation, we observed a larger zone of inhibition against *P. aeruginosa* and *S. aureus* subsp. *aureus* compared to the previous reported studies [51]. This attribute of antimicrobial activity can assist *S. clausii* strains in establishing dominance over existing pathogens in the gut environment, thereby preventing infection.

Furthermore, an exploration of potential antimicrobial compounds was undertaken using BAGEL 4.0 [38], a tool that predicted the presence of bacteriocin biosynthetic gene clusters affiliated with the sactipeptide and BsaA2 categories within the four genomes. Sactipeptides represent a group of bacteriocins distinguished by the presence of a characteristic thioether bridge, known as the sactionine bond. This particular bridge is post-translationally incorporated, and its existence plays a critical role in facilitating the antimicrobial properties of sactipeptides [52]. The existence of these genes within B619/R, B603/Nb, B106, and B637/Nm suggests their potential to harbor antimicrobial capabilities, thereby assisting in establishing themselves within the gastrointestinal tract and contributing to the exclusion of harmful pathogens. Furthermore, this genetic configuration offers valuable insights into the hypothetical mechanism through which these strains could combat disease-causing pathogens, such as *Helicobacter pylori*, *Gardenerella vaginalis*, *Listeria monocytogenes*, *Clostridium difficile*, *Staphylococcus pneumoniae*, and *Streptococcus aureus*, as previously outlined by Cotter et al. [53]. The identification of a biosynthetic gene cluster associated with BsaA2, a constituent of the epidermin-like lantibiotics family with recognized bactericidal properties, was also observed within B619/R, B603/Nb, B106, and B637/Nm. Notably, the prevalence of the BsaA2 gene has been extensively documented within various species of *Staphylococcus* [54].

The antimicrobial compounds produced by *S. clausii* have been identified and characterized in previous studies. The lantibiotic clausin, which was initially discovered in the cell-free supernatants of *S. clausii* O/C, has been found to be effective against various Gram-positive bacteria, including *C. difficile* [55]. Interestingly, whey fermentation by *S. clausii* has also revealed the production of antimicrobial substances active against both Gram-negative and Gram-positive species [56], highlighting the importance of starting substrates for antimicrobial synthesis. Additionally, another significant example of indirect antimicrobial activity was reported by Ripert et al. [57]. This study described a serine protease produced during the sporulation phase by *S. clausii* O/C, which can neutralize the cytotoxic effects of toxigenic strains of *C. difficile* and *B. cereus*.

### 3.4. Health-Promoting Effects of S. clausii Strains

The beneficial characteristics exhibited by the four strains, including protease, lipase, β-galactosidase, antioxidant activity, and the ability to lower cholesterol levels, are illustrated in Table 1. All four strains exhibited protease activity. Lipase activity was observed in B619/R, B603/Nb, and B106. However, this activity was not observed in B637/Nm. Protease and lipase activity are known to enhance the nutritional amenability of the food consumed. β-galactosidase production has been documented for some *S. clausii* strains. Mazzantini et al. reported lower levels of this enzyme production in Enterogermina strains [18]. In the present study, β-galactosidase production was displayed by all four strains, and the administration of such β-galactosidase-producing probiotics can be advantageous for people affected by lactose intolerance. The maximum antioxidant activity was shown by B603/Nb (95%), followed by B619/R (80%). B637/Nm exhibited the lowest antioxidant activity (68%). The antioxidant activity indicates their potential to shield the host from damage caused by reactive oxygen species (ROS) that are linked to the development of several chronic illnesses. The antioxidant capacity of *S. clausii* strains has limited supporting evidence, with available studies mainly concentrated on cell lines and murine models. Notably, in a rat model of uremia, the administration of UBBC07 showed promising results in alleviating the oxidative response induced by acetaminophen, evidenced by an increase in the levels of glutathione, superoxide dismutase, and catalase [58]. Additionally, *S. clausii* has been demonstrated to suppress the production of reactive oxygen species in the Caco-2 cell line, specifically in response to rotavirus infection [59]. The evidence concerning the cholesterol reduction activity of *S. clausii* remains scarce, with the available references not directly addressing this particular aspect. It is noteworthy that the strains utilized in the present study demonstrated cholesterol-lowering activity. B637/Nm showed ~60% cholesterol-reducing ability, whereas B619/R, B603/Nb, and B106 could reduce 46%, 55%, and 52% cholesterol in SIF, respectively. Probiotics, in general, have been studied for their potential to modulate lipid profiles and reduce cholesterol levels [60]. However, to ascertain the specific effects of *S. clausii* on cholesterol reduction, further investigation is required.

As evident from the above findings, *S. clausii* strains displayed remarkable potential as a probiotic with potential benefits for gut health, and to fully harness these benefits, it would be essential to delve into the mechanisms and molecular factors involved in their adhesion to the gut epithelium. Gaining insights into adhesion mechanisms would facilitate the development of probiotic formulations that are both more efficient and tailored to specific strains, thereby promoting gut health and overall well-being in individuals more effectively [61].

**Table 1:** Probiotic properties of *S. clausii* strains.

Properties	*S. clausii* Strains			
	B619/R	B603/Nb	B106	B637/Nm
**Identification**				
% 16S rRNA gene identity with *S. clausii* DSM 8716	99.85	99.85	99.85	99.87
Average Nucleotide Identity (ANI) (%)	95.45	95.42	95.44	95.44
Digital DNA-DNA Hybridization (DDH) (%)	62.5			
**Physiological properties ^a^**				
Optimum Temperature (°C)	30 (22–45)	30 (22–45)	30 (22–45)	30 (22–45)
Optimum pH	10 (7–10)	10 (7–10)	8 (7–10)	10 (7–10)
Growth in presence of NaCl (%)	4 (0–10%)	2 (0–10%)	4 (0–10%)	4 (0–10%)
**Survival in Gastrointestinal fluids ^b^**				
Survival (%) in SGF (120 min)	88 ± 8	57 ± 3	94 ± 19	88 ± 4
Survival (%) in SIF (180 min)	70 ± 1	36 ± 0	66 ± 2	71 ± 2
**Antibiotic resistance profile** ^c^ (*strains were resistant to the listed antibiotics)	Streptomycin, Neomycin, Clarithromycin, Metronidazole, Chloramphenicol	Novobiocin, Rifampicin, Clarithromycin, Metronidazole, Amoxicillin, Chloramphenicol	Chloramphenicol, Clarithromycin, Metronidazole	Tetracycline, Chloramphenicol Clarithromycin, Metronidazole, Amoxicillin
**Disease Preventing traits**				
Antimicrobial activity against pathogens ^d^	*P.* *aeruginosa* (14.5 ± 0.7 mm)	*P. aeruginosa* (27 ± 1.4 mm), *S. aureus* subsp. *aureus* (20 ± 2.1 mm)	*P. aeruginosa* (11 ± 0.8 mm), *E. faecalis* (24.5 ± 0.7 mm), *and* *S. aureus* subsp. *aureus* (23 ± 2.6 mm)	*P. aeruginosa* (15.6 ± 1.1 mm), *E. faecalis* (10.6 ± 1.1 mm), *and* *S. aureus* subsp. *aureus* (18 ± 2.8 mm)
**Health Promoting traits**				
Protease activity ^e^	+	+	+	+
Lipase activity ^f^	+	+	+	-
β-galactosidase activity ^g^	+	+	+	+
Antioxidant activity (%) ^h^	80 ± 2	95 ± 1	73 ± 3	68 ± 1
Cholesterol-reducing ability (%) in SIF ^i^	46 ± 7	55 ± 1	52 ± 1	60 ± 2
Exo-polysaccharide production (mg/L) ^j^	467 ± 10	463 ± 9	625 ± 11	532 ± 5

Note: ANI: Average Nucleotide Identity; DDH: Digital DNA-DNA hybridization; SGF: Simulated Gastric Fluid; SIF: Simulated Intestinal Fluid. The numbers in the parenthesis indicate the range of the parameters that allowed the growth of the cultures. ^a^: Optimum growth conditions, in terms of pH, temperature, and salinity, were recorded by measuring the OD of the strains after 24 h at 600 nm. ^b^: The tolerance of *S. clausii* spores to harsh GIT conditions evaluated by assessing the viability of each of the *S. clausii* strain after exposure to SGF and SIF, illustrated in terms of % survival. ^c^: The antibiotic susceptibility/resistance of *S. clausii* strains was evaluated using E-test strips and the data pertaining to resistance are provided. ^d^: The ability of *S. clausii* strains to inhibit the growth of pathogens evaluated using the agar overlay method is illustrated in terms of the diameter of the zone of inhibition observed against the test pathogens. ^e^: The proteolytic activity of the strains evaluated by checking for the zone of clearance on skimmed milk agar is illustrated in terms of the sign ‘+’, which indicates positive activity, while ‘-’indicates negative activity. ^f^: The lipolytic activity of the strains evaluated by checking for the zone of clearance on tributyrin agar is illustrated in terms of the sign ‘+’, which indicates positive activity, while ‘-’ indicates a negative activity. ^g^: The β-galactosidase activity of the strains evaluated by checking for color changes on CLED agar is illustrated in terms of the sign ‘+’, which indicates positive activity, while ‘-’ indicates a negative activity. ^h^: Antioxidant activity of the strains investigated by DPPH-free radical scavenging activity. ^i^: Cholesterol-reducing ability represented in terms of the percentage of cholesterol assimilated by strains. ^j^: Exo-polysaccharide production estimated by the Phenol–Sulphuric acid method.

### 3.5. Adhesion of S. clausii Strains to Human Colonic Epithelial Cells

Effective colonization of the human intestine is vital for probiotic organisms to exert their beneficial effects [62]. Adhesion to the mucosal surface is a desirable trait for colonization and persistence in the gastrointestinal tract [63]. However, the adhesion potential of spore-forming bacteria of the genus *Shouchella* has been relatively understudied. Therefore, we conducted a thorough investigation into the ability of *S. clausii* strains to adhere to intestinal cells and ECM components, along with the evaluation of their cell surface properties, such as hydrophobicity and aggregation, as well as the genetic markers that contribute to adhesion.

The colonic adenocarcinoma cell lines HT-29 and Caco-2 are commonly utilized as model systems for studying the interaction between probiotics and human intestinal cells [64]. These cell lines are derived from human intestinal epithelium and serve as valuable *in vitro* models. These cell lines have morphological similarities to mature enterocytes as well as functional similarities in terms of the expression of enzymes, receptors, and transporter proteins present in the normal human intestinal epithelium [65,66]. Employing both Caco-2 and HT-29 cell lines for studying bacterial adhesion allows for a more comprehensive understanding of the interactions between bacteria and the intestinal barrier. The Caco-2 cell line provides insights into the absorptive and defensive properties of the intestinal mucosa, while the HT-29 cell line offers a different perspective on bacterial adhesion. Incorporating multiple cell lines allows for the consideration of variations, facilitates result comparison, and validates the obtained findings [67,68].

**Figure 1:**
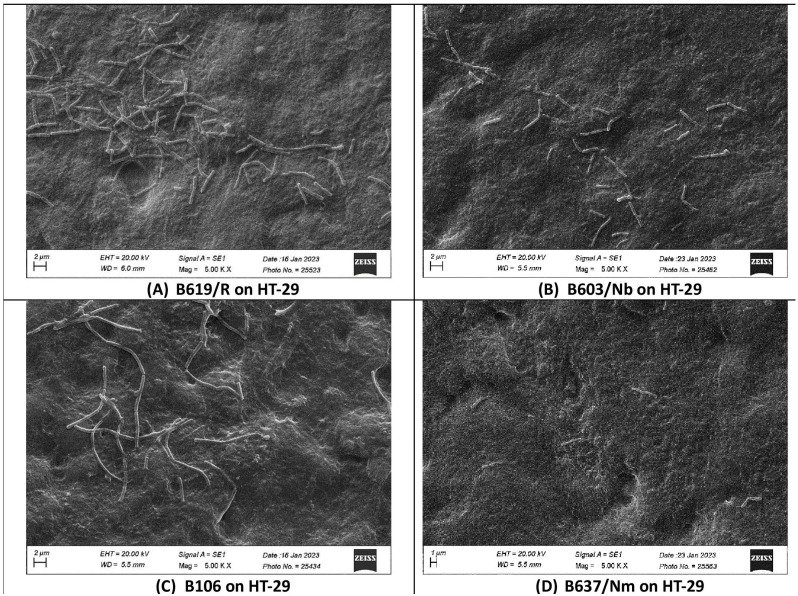
Scanning electron micrographs of differentiated HT-29 cell monolayers with adhering *S. clausii* strains.

**Figure 2:**
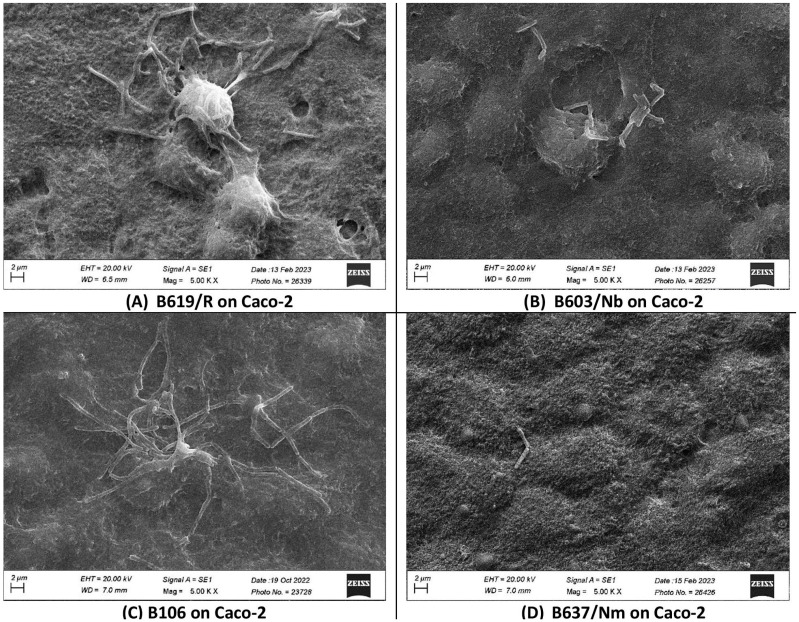
Scanning electron micrographs of differentiated Caco-2 cell monolayers with adhering *S. clausii* strains.

In the present study, we investigated the quantitative binding of *S. clausii* strains to HT-29 and Caco-2 cell lines using direct microscopic examination under scanning electron microscopy. Even after extensive washing with PBS, a significant proportion of bacterial cells remained attached to the monolayer, proving that the adhesion was not merely nonspecific physical entrapment. The microscopically observed adhesion efficiency was further validated by enumerating the adherent bacteria. The adhesive phenotype was evaluated as per the findings reported by Haeri et al. [66]. Bacterial strains were categorized as poorly adhesive if < 20 bacterial cells adhered per 100 animal cells, moderately adhesive if 21–50 bacteria adhered per 100 animal cells, and strongly adhesive if >51 bacteria adhered per 100 animal cells [66]. The results indicated variability in the adhesion capacity of *S. clausii* across different strains. B619/R, B603/Nm, and B106 displayed higher adhesion to the HT-29 cell line, ranging from 211–360 bacterial cells adhering per 100 HT-29 cells, while B637/Nm showed poor adhesion, with only 11 cells adhering per 100 HT-29 cells (Table 2; Figure 1). A similar pattern was observed with the Caco-2 cell line (Table 2; Figure 2). The strong adhesion exhibited by *S. clausii* strains would increase their opportunity to interact with the host, potentially leading to temporary colonization and prolonged transit time in the gut, thereby facilitating the exertion of their intended beneficial effects.

While only two reports have previously documented the adhesion potential of *S. clausii*, they described the moderate adhesive phenotypes of strain CSI08 and strains derived from Enterogermina to HT-29-MTX and Caco-2 [12,69]. In contrast, our study reports the high adhesive nature of *S. clausii* strains on HT-29 as well as Caco-2 cell lines. To gain a more comprehensive understanding of the strain-dependent variation in the adhesion pattern, a detailed investigation into the adhesion potential was undertaken. This investigation was particularly relevant because bacterial adhesion to intestinal surfaces is often initiated by non-specific physical interactions, especially hydrophobic interactions [70]. Subsequently, specific cell wall components, such as fimbriae or pili, adhesins, mucus-binding proteins, fibronectin-binding proteins, or surface layer proteins, play a crucial role in enabling bacteria to more effectively colonize epithelial cells. Additionally, lipoteichoic acid or exopolysaccharides produced by bacteria are known to facilitate adhesion to host epithelial cells [67]. By exploring the ECM binding abilities, cell surface properties, and genomic analysis of the *S. clausii* strains, this investigation aims to shed light on the mechanisms underlying their strain-specific adhesion capabilities to intestinal surfaces.

### 3.6. Competitive Exclusion of S. Typhimurium Adhesion by S. clausii to HT-29 Cells

Competitive exclusion is a mechanism by which probiotic bacteria inhibit the growth and colonization of pathogens in the human body by competing for adhesion sites on the intestinal epithelial cells [71]. In the present study, the exclusion of *S.* Typhimurium adhesion to HT-29 cells by *S. clausii* strains was evaluated. All four strains of *S. clausii* exhibited the ability to inhibit the adhesion of *S.* Typhimurium to HT-29 cells. Among them, B619/R exhibited the highest exclusion of *S.* Typhimurium from HT-29 cells, accounting for 45%, while B106 exhibited the lowest exclusion ability of 10% compared to the other strains (Table 3; Figure S1). Interestingly, there are no published reports on the pathogen exclusion ability of *S. clausii*.

The precise mechanisms by which probiotic bacteria prevent pathogen colonization are not clearly explained, but it is widely known that competitive exclusion plays a key role. In this study, the most probable mechanism by which *S. clausii* strains excluded *S.* Typhimurium from HT-29 cells could be either by competing for adhesion sites on intestinal epithelial cells, thus inhibiting the colonization of *S.* Typhimurium, or by co-aggregating with the pathogen, thereby effectively preventing its adhesion.

### 3.7. In Vitro Binding of S. clausii Strains to ECM Components

The epithelial cells lining the GIT are covered with a mucus layer, acting as the primary barrier for ingested microorganisms. Adherence to this mucus matrix is believed to be crucial for the colonization of the gut. Although the ECM surrounds epithelial cells in a complex structure, molecules from the ECM, such as collagen-I and fibronectin, can pass from the epithelium into the mucus. In addition, mucosal injury can expose the ECM, allowing for microbial colonization and infection. This is the reason why lactobacilli binding to ECM proteins may aid pathogen competitiveness and eviction [15].

In an attempt to determine whether the binding of *S. clausii* strains to intestinal epithelial cell lines HT-29 and Caco-2 correlated with the binding of molecules present in the mucus or the ECM, their binding to mucin, fibrinogen, and collagen immobilized on the surface of microtiter plates was measured. All of the tested strains displayed binding to ECM components at detectable levels. Strains B619/R, B603/Nb, B106, and B637/Nm displayed comparable binding to mucin, while differential binding was observed in the case of fibrinogen and collagen. Strains B619/R and B637/Nm exhibited significant binding to fibronectin, while strain B603/Nb displayed strong affinity for collagen. Notably, among the four strains, B637/Nm displayed significantly low binding to collagen (Table 4). These findings clearly indicate that the binding affinity of *S. clausii* with different ECM components is an independent event and thus supports the strain-specific nature of probiotic adhesion properties. Such strain-specific affinity towards ECM components has been documented in other studies. Differential binding capabilities with ECM components have been observed by *L. casei* strains isolated from different sources, with some strains showing intermediate binding and others displaying the lowest binding ability to the same ECM components [15,72]. These observations underscore the diversity and complexity of bacterial interactions with ECM components, which may influence their potential to colonize and persist in the gut environment.

The ability of *S. clausii* strains to adhere to a specific element of the extracellular matrix (ECM), namely mucin, has been documented in studies by Ahire et al. and Mazzantini et al. [16,18]. *S. clausii* UBBC07 exhibited a notably low binding affinity of 0.065 to mucin. However, our study reveals that the binding affinities of all four *S. clausii* strains to mucin were considerably higher (>0.2), characterizing our strains as potentially adherent strains. These findings are indicative of a probable mechanism for establishing close interactions with host tissues and cells.

### 3.8. Cell Surface Properties of S. clausii Strains

A potential probiotic strain’s cell surface characteristics significantly contribute to evaluating its likely positive effects on the host. Among non-specific interactions, hydrophobic interaction and aggregating interaction between surface macromolecules have been regarded as the primary indicators for assessing the colonization capacity of probiotics [73]. Electrostatic, van der Waals, hydrogen-bonding, and hydrophobic interactions mediate the earliest non-specific physicochemical interactions of microorganisms. Bacterial adhesion to hydrocarbons (BATH) is a technique that assesses how hydrophobic potential probiotic strains operate in a liquid-liquid interference system when exposed to aliphatic or aromatic hydrocarbons [74].

The surface hydrophobicity for *S. clausii* strains was analyzed by measuring adhesion to solvents such as chloroform, ethyl acetate, hexadecane, xylene, and toluene (Table 5). Strain B637/Nm exhibited the maximum percent surface hydrophobicity (~60–67%) in all the tested non-polar solvents, followed by B603/Nb (~ 19–44%) and B619/R (~ 3–32%). The minimum surface hydrophobicity was exhibited by B106 (~4–11%). Strains B619/R, B606/Nb, and B106 exhibited low hydrophobicity levels (H% < 50%), indicating their preference for the aqueous phase. In contrast, strain B637/Nm displayed high cell surface hydrophobicity (H% > 60%). However, despite its high hydrophobicity, strain B637/Nm showed weak adhesion to the HT-29 and Caco-2 cell lines (Table 2). In a previous study, Ahire et al. demonstrated that *S. clausii* strain UBBC07 had a less hydrophobic nature with xylene, showing a hydrophobicity value of 22% [16]. According to a few studies, a correlation exists between lactobacilli’s capacity to adhere to surfaces and their surface hydrophobicity [75], although others found no such correlation [76]. In agreement with this, no correlation between the strains hydrophobicity and binding abilities was discovered in the present study. Strains B619/R, B603/Nb, and B106 had a comparatively low hydrophobic surface and good adhesion, whereas the hydrophobic strain B637/Nm exhibited low adhesion to the cell lines. More investigation is required to define the function of cell surface properties in bacterial adhesion.

Cellular aggregation implicates the ability to precipitate similar kinds of cells. The auto-aggregation of probiotic cells is reported to be mediated by cell surface proteins and a few soluble proteins. This form of interaction is advantageous for the effective colonization and communication of probiotics with the host tissue. Additionally, it strengthens the antimicrobial potential of probiotics and helps them compete with and exclude pathogens from the intestinal mucosa. Both hydrophobicity and aggregation ability are known to affect the specific attachment of probiotics to the host epithelial mucosa [77]. Probiotic bacteria need to achieve an adequate mass through aggregation to manifest beneficial effects. Consequently, the ability of probiotics to aggregate is a desirable property. Organisms that can aggregate with other bacteria, such as pathogens, may have an advantage over non-co-aggregating organisms, which are more easily removed from the intestinal environment [77].

In the present study, *S. clausii* strain B637/Nm demonstrated a high auto-aggregation of 75.90% (Table 6). B619/R (43%), B603/Nb (35%), and B106 (45%) displayed moderate auto-aggregation percentages. According to the study by Jeon et al., *S. clausii* ATCC 700160 exhibited the highest auto-aggregation of 85.10% after 4 h of incubation [17].

The co-aggregation of probiotics with pathogenic bacteria represents a probable mechanism by which probiotics prevent the attachment of pathogens to the intestinal surface, thereby inhibiting their colonization in the human GIT [78]. Hence, in the present study, the co-aggregation potential of *S. clausii* strains was evaluated against the pathogens *E. coli*, *S. aureus* subsp. *aureus*, *E. faecalis*, *S. dysenteriae*, *K. pneumoniae*, *P. aeruginosa*, *E. aerogenes*, and *S.* Typhimurium. All the strains were able to exhibit co-aggregation with the test pathogens. The strains B619, B603/Nb, and B106 showed moderate co-aggregation potential (~42–45%) against *S. dysenteriae*, *E. coli,* and *P. aeruginosa*, respectively. B637/Nm, when compared to other strains, displayed a higher co-aggregation potential of ~ 30–44% with most of the tested pathogens (Table 6), indicating its ability to form an effective barrier and prevent the adhesion of enteric pathogens. There has been no study reported regarding the co-aggregation potential of *S. clausii* against pathogens.

Numerous studies have provided evidence that increased auto-aggregation and hydrophobicity are correlated and associated with enhanced adhesion [34]. Our study deviated from this observation, with no correlation observed between adhesion and hydrophobicity/auto-aggregation. A report by Krausova et al. [74] indicated no correlation between hydrophobicity and adhesion capacity, while Chaffanel et al. [79] demonstrated no correlation between auto-aggregation and the adhesion ability of probiotic bacteria. This suggests other factors beyond hydrophobicity alone can influence bacterial adhesion. Factors such as the expression of specific adhesins, fimbriae, or other surface structures may play important roles in mediating bacterial attachment, independent of overall cell surface hydrophobicity. The composition and conformation of the bacterial cell surface can be complex, and hydrophobicity is just one of many physicochemical properties that impact adhesion. Strain-specific differences in these surface characteristics likely explain the variable relationship observed. The lower adhesion of the highly hydrophobic strain B637/Nm suggests that hydrophobicity alone does not determine adhesion potential. The higher adhesion of strains B619/R, B106, and B603/Nm with lower hydrophobicity indicates other adhesion mechanisms are likely more important for these particular strains. The correlation between hydrophobicity and adhesion can be strain-dependent.

### 3.9. Genomic Insights into the Adhesion Potential of S. clausii Strains

Genome mining revealed a comprehensive set of adhesion-related genes (Table S1) in all four *S. clausii* strains. The predicted fibronectin-binding protein (FnBP) and collagen adhesion protein indicated the strains’ ability to bind to fibronectin and collagen, common extracellular matrix (ECM) proteins in the human colon and ileum. Previous studies have reported a correlation between fibronectin binding and bacterial adherence to intestinal cells *in vitro*, as well as *in vivo* [80]. *S. clausii* strains lacked the mucin-binding proteins; however, the sortase-dependent protein LspA was detected, which has been reported to mediate the bacterial adhesion to human epithelial cells and mucus [81]. *S. clausii* strains harbored a functional sortase A, which serves a housekeeping function by anchoring surface-dependent proteins with an LPXTG recognition sequence, suggesting its involvement in interactions with intestinal epithelial cells or mucosal components [82]. Additionally, several moonlighting proteins, such as GAPDH, Enolase, EF-Tu, EF-G, Triosephosphate isomerase, GroEL, DnaK, pyruvate kinase, Inosine 5′-monophosphate dehydrogenase (IMPDH), Glutamine synthetase, and Glucose-6-phosphate isomerase (GPI), were identified in *S. clausii* genomes. These moonlighting proteins fulfill diverse adhesive functions, which encompass adhesion to host epithelia and host components, such as ECM and plasminogen [83].

Glyceraldehyde-3-phosphate dehydrogenase (GAPDH), an anchorless, multifunctional protein on the bacterial surface, has been found to interact with various host components, such as plasminogen, cytoskeleton proteins, and ECM proteins like fibronectin [84]. The cell wall association of enolase and GAPDH in probiotic bacteria showed reversible and pH-dependent cell wall association in probiotic bacteria, impacting their adhesion to cell surfaces [85]. These properties likely influence probiotic/host interactions, with the acidic pH of the intestinal mucosa favoring attachment to bacterial surfaces, while a neutral or slightly alkaline pH induces detachment into the intestinal content. Recent studies have demonstrated the ability of GAPDH from *L. plantarum* to bind to human colonic mucin [86]. Similarly, Antikainen et al. found that among the proteins present in the surface-associated proteome of *L. crispatus*, only enolase could bind to laminin and collagen [87]. Furthermore, surface-associated forms of bacterial GAPDH and enolase have been implicated in plasminogen (Plg)/plasmin binding and activation [88].

EF-Tu has also been reported to have moonlighting functions associated with bacterial adherence to ECM molecules, such as glycosaminoglycans, fibronectin, and host cells [89]. Granato and colleagues conducted research on the probiotic strain *L. johnsonii* NCC 533 (La1) and identified the presence of EF-Tu on its surface as they sought to identify molecules involved in attachment to intestinal epithelial cells and mucin. Additionally, they found that recombinant La1 EF-Tu (rEF-Tu) could bind to intestinal epithelial cell lines (Caco-2 and HT-29) and human gut mucin. The adhesion of EF-Tu to epithelial cells or mucus was found to be pH-dependent, similar to other Mub mucus-binding proteins found in other probiotic strains. Interestingly, rEF-Tu was shown to stimulate the release of IL-8 from HT-29 cells in the presence of soluble CD14, suggesting that EF-Tu might play a role in gut homeostasis through its interaction with the intestinal mucosa [89]. GroEL, a HSP60 homologue, has been extensively studied as a moonlighting protein contributing to adhesion to mucin [90]. Moreover, the *S. clausii* genome was found to harbor genes responsible for EPS biosynthesis, notably the glycosyltransferases epsA, epsC, and epsD. These gene predictions suggest a potential function in aiding adhesion to intestinal mucus. To confirm these hypotheses, *in vitro* trials were conducted, revealing noticeable EPS production by the test strains (Table 1). The above findings shed light on the various adhesion-related mechanisms employed by *S. clausii* strains, providing valuable insights into their potential as probiotics in the gastrointestinal environment.

Our study was found to be in congruence with the published report for *S. clausii*. The study by Khatri et al. involved a composite genome analysis of ENTPro, wherein genes encoding mucus-binding protein, collagen-binding protein, fibronectin-binding protein, Sortase, Flagellin proteins, and triosephosphate isomerase were found to be the major proteins involved in adhesion [49].

### 3.10. Comparative Analysis of Adhesion Proteins in S. clausii Strains

Comparing the adhesion proteins in B619/R, B106, B603/Nb, and B637/Nm revealed significant similarities (Table S1). Notably, strain B637/Nm lacked the gene encoding the cell wall surface anchor family protein (LPxTG motif), which plays a significant role in interactions with intestinal epithelial cells and/or mucus components. Surface proteins in bacteria are anchored to the cell wall peptidoglycan through a mechanism involving a C-terminal sorting signal with an LPXTG motif. This process involves the action of a membrane-anchored transpeptidase called sortase, which cleaves proteins at the LPxTG motif and forms a thioester bond with the conserved cysteine of sortase. The newly liberated C-terminus of the protein is then linked to the bacterial peptidoglycan, effectively tethering the surface protein to the cell wall [91].

The surface proteins dependent on sortase for anchoring, including mucin-binding proteins (Mub and Msa) and LspA, are referred to as sortase-dependent proteins. These proteins contain both an N-terminal signal peptide and a C-terminal sorting signal, typically comprising a conserved LPxTG motif, a hydrophobic domain, and a positively charged residue tail. In strain B637/Nm, the absence of the LPXTG motif prevents sortase-mediated cell wall linkage and subsequently impairs further interactions.

Proteins that adhere to mucus generally share common characteristics, such as a signal peptide, a C-terminal cell wall-anchoring motif (usually LPXTG-like), multiple repeated domains with putative adhesion functions, and regions with unknown functions. The presence of these proteins on the surface of probiotic bacteria could be crucial for their persistence in the gut epithelium. This point has been suggested in the strain *L. salivarius* UCC118, an isogenic sortase mutant lacking LPXTG proteins on the surface, which showed reduced adhesion capabilities to human cell lines Caco-2 and HT-29 [92]. This highlights the significance of sortase-dependent proteins in mediating bacterial adhesion to host cells and mucus in the gut environment.

### 3.11. In Silico Characterization of the Cell Surface Protein of S. clausii Strains

*In silico* characterization of the cell wall surface anchor family protein (LPxTG motif) revealed that it shares 100% similarity among three strains (B619/R, B106, and B603/Nb). This protein was found to be acidic due to a pI value of less than 7. The computed Extinction coefficient (ε) was determined to be 22,920 M^−1^ cm^−1^, indicating the presence of a high concentration of Cys, Trp, and Tyr residues. The aliphatic index, measured at 80.61, suggested that this protein is stable over a wide range of temperatures. Additionally, the calculated GRAVY value of −0.521 indicated that the protein is nonpolar. Moreover, the estimated instability index (II) of 26.25 indicated the relative stability of the protein.

Overall, the *in silico* characterization highlights the interesting properties of this cell wall surface anchor family protein, making it a potentially significant molecule for further research and study.

#### 3.11.1. Predicted 3D-Structure of the Cell Surface Protein

**Figure 3:**
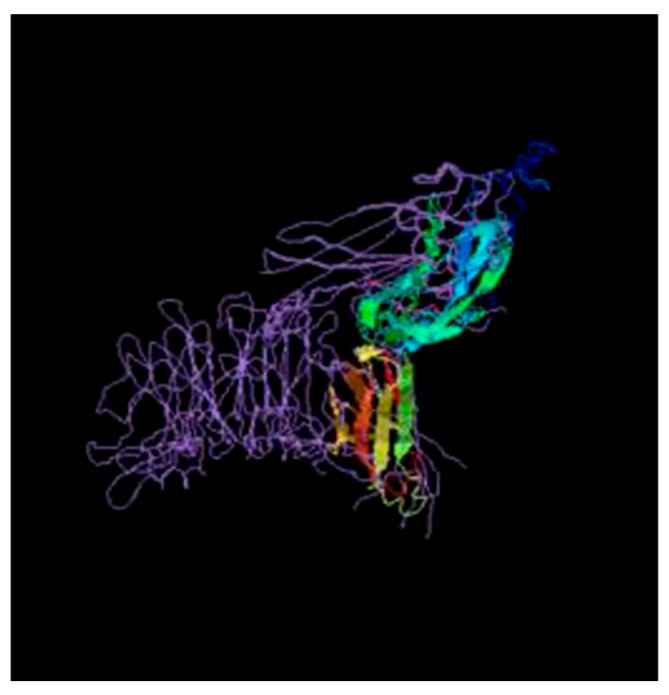
Modeled structure of the cell wall surface anchor family protein (LPxTG motif).

The 3D structure of the cell wall surface anchor family protein (LPxTG motif) (Figure 3) was elucidated by Phyre 2.0. Two hundred forty-two (242) residues (86% of the sequence) were modeled, with 98.4% confidence by the single highest scoring template, indicating the reliability of the model.

#### 3.11.2. Docking of the Cell Wall Surface Anchor Family Protein (LPxTG Motif) with Gut Mucins

**Figure 4:**
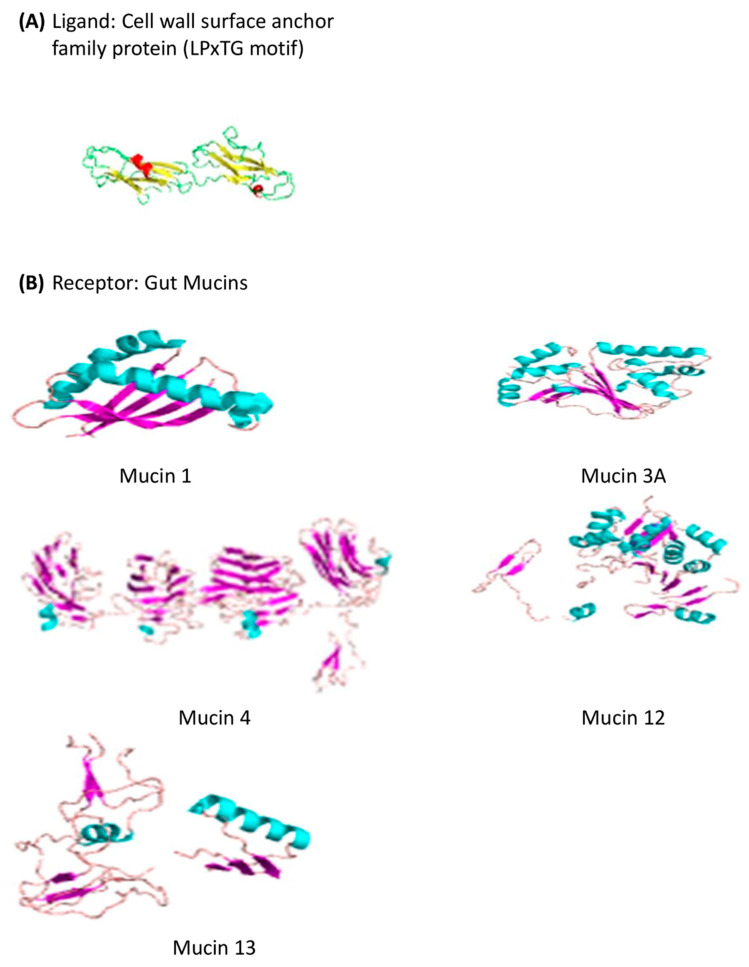
Modeled structures of adhesion protein and gut mucins.

**Figure 5:**
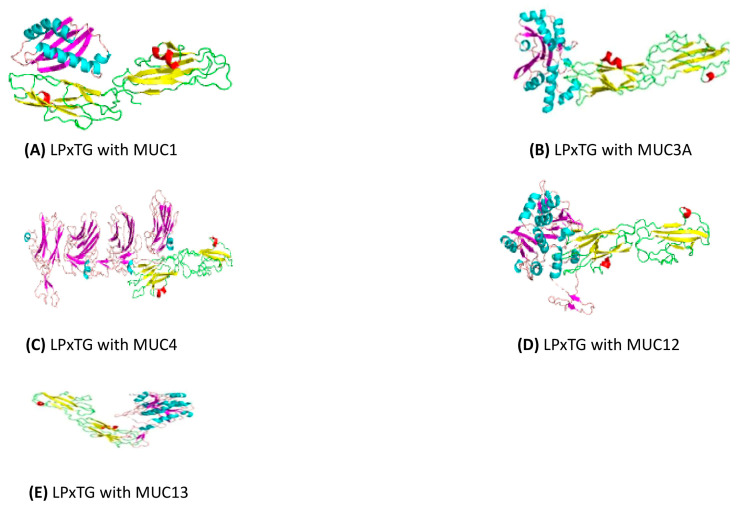
Molecular docking studies of adhesion protein of *S. clausii* with gastrointestinal mucins.

The Cluspro protein-protein docking strategy was employed for *in silico* docking to predict the binding affinity between *S. clausii’s* cell wall anchor protein and gastrointestinal mucins (MUC1, MUC3A, MUC4, MUC12, and MUC13) (Figure 4). The adhesion of probiotic bacteria to mucin is a critical factor contributing to their persistent beneficial effects in the gut. Mucins, which are glycoproteins present in the mucus layer of the gastrointestinal tract, provide binding sites for both pathogens and commensal bacteria [93]. Through this type of investigation, molecular-level insights into bacterial adhesion mechanisms could be gained, and potential targets for enhancing probiotic effects might be identified. The results of these studies demonstrated their strong binding affinity and interaction with each other. Notably, among the potential interactions, the interactions between LPxTG and MUC4, followed by MUC12, showed the highest affinity and lowest energy, with binding energies of −1317.1 kcal/mol and −1122.9 kcal/mol, respectively, among all the complexes that were docked (Table 7, Figure 5). This type of interaction can be considered the primary factor influencing the adhesion of *S. clausii* to mucus.

This is the first report on an *in silico* adhesion assay for *S. clausii*. Such docking studies have been used for the evaluation of the adhesion potential of *Lactobacillus acidophilus*, *Lactiplantibacillus plantarum*, *L. brevis*, *Limosilactobacillus fermentum*, and *Pediococcus acidilactici* [93,94]. Docking studies and *in vitro* adhesion studies demonstrated the potential of *S. clausii* strains as probiotics capable of adhering to the gastrointestinal mucosa.

## 4. Conclusions

This study presents novel insights into the adhesion potential and cell surface properties of *Shouchella clausii* strains isolated from healthy human volunteers. *In vitro* tests evaluated the strains’ adhesion to human colonic cells, revealing strain-specific differences. B619/R, B603/Nb, and B106 displayed strong adhesion, while B637/Nm showed weak binding, highlighting the strain-specific capacity of *S. clausii* to interact and attach to intestinal cells. Genomic data validated the *in vitro* findings, predicting adhesion and moonlighting proteins. *In silico* protein docking affirmed strong *S. clausii*-gut mucin interactions. Moreover, no correlation existed between cell surface properties and the colonization potential of the strains belonging to the same species. The study identifies specific probiotic traits unique to the *S. clausii* strains and underscores the importance of using multiple strains for an effective probiotic formulation. These findings contribute to our understanding of the interactions between probiotic bacteria and the host, providing insights into their potential beneficial effects in the gastrointestinal tract.

## Figures and Tables

**Table 2:** Assessment of the ability of *S. clausii* to adhere to epithelial cells (HT-29 and Caco-2).

*S. clausii* Strain	Number of *S. clausii* Cells Adhering/100 HT-29 Cells	Number of *S. clausii* Cells Adhering/100 Caco-2 Cells
B619/R	360 ± 14	182 ± 10
B603/Nb	114 ± 6	106 ± 9
B106	211 ± 23	210 ± 30
B637/Nm	11 ± 3	19 ± 1

The epithelial (HT-29 and Caco-2) smear post bacterial adhesion was examined under scanning electron microscopy.

**Table 3:** Inhibition of the adhesion of *S.* Typhimurium to HT-29 by *S. clausii* strains.

*S. clausii* Strain	% Exclusion of *S.* Typhimurium from HT-29 Cell Line by *S. clausii* Strain
B619/R	37 ± 3
B603/Nb	19 ± 1
B106	7 ± 1
B637/Nm	18 ± 1

% Exclusion refers to the percentage of *S.* Typhimurium excluded from adhering to HT-29 cells in the presence of *S. clausii* strains.

**Table 4:** Binding of *S. clausii* strains to different components of the Extracellular Matrix (ECM).

*S. clausii* Strain	Binding to ECM Components (O.D. _600 nm_)		
	Mucin	Fibrinogen	Collagen
B619/R	0.250 ± 0.008	0.203 ± 0.008	0.175 ± 0.004
B603/Nb	0.206 ± 0.039	0.105 ± 0.002	0.202 ± 0.003
B106	0.241 ± 0.032	0.154 ± 0.005	0.163 ± 0.003
B637/Nm	0.247 ± 0.009	0.204 ± 0.002	0.048 ± 0.002

Note: Strains were classified as strongly adherent (A600 nm ≥ 0.3), weakly adherent (0.1 ≤ A600 nm ≥ 0.3), or nonadherent (A600 nm ≤ 0.1), as described previously [15].

**Table 5:** Estimation of cell surface hydrophobicity of *S. clausii* strains.

Strains of *S. clausii*	Hydrophobicity (%)				
	Chloroform	Ethyl Acetate	Toluene	Xylene	n-Hexane
B619/R	19.1 ± 7.3	19.3 ± 0.5	32.7 ± 1.8	12.1 ± 0.1	3.1 ± 1.2
B603/Nb	17.6 ± 0.2	20.4 ± 0.3	44.8 ± 0.5	33.1 ± 1.3	19.7 ± 0.7
B106	11.0 ± 0	4.4 ± 1.5	11.2 ± 0.2	4.2 ± 0.1	4.6 ± 1.2
B637/Nm	27.3 ± 3.6	57.4 ± 0.5	67.4 ± 1.1	61.7 ± 0.4	60.4 ± 0

**Table 6:** Percentage of auto-aggregation and co-aggregation of *S. clausii* strains with pathogens.

Strains of *S. clausii*	Auto-aggregation (%)	% Co-Aggregation of *S. clausii* Strains with Pathogens after 4 h							
		*E. coli*	*S. aureus* subsp. *aureus*	*E. faecalis*	*S. dysenteriae*	*K. pneumoniae*	*P. aeruginosa*	*E. aerogenes*	*S.* Typhimurium
B619/R	43.1 ± 4.4	14.8 ± 1.7	38.1 ± 3.6	16.2 ± 1.0	42.1 ± 1.3	10.4 ± 1.1	12.4 ± 1.5	37.3 ± 4.4	24.3 ± 3.6
B603/Nb	35.8 ± 2.4	45.5 ± 0.2	39 ±0.5	28 ± 0.01	28.5 ± 5.4	28.4 ± 0.8	41.2 ± 9.3	27.1 ± 2.1	32.1 ± 0.3
B106	45.7 ± 0.4	30 ± 1.2	42.9 ± 0.8	28 ± 3.4	26.3 ± 3.3	21.6 ± 2.1	42.5 ± 3.4	26.9 ± 3.1	24.5 ± 0.6
B637/Nm	75.9 ± 0.9	33 ± 0	43.9 ± 0.1	34.1 ± 0.08	41.6 ± 2.2	30.3 ± 1.2	35.8 ± 0.08	40.0 ± 0.07	29.3 ± 1.4

**Table 7:** Energy score of *S. clausii*’s adhesion protein with gut mucins.

Docking Complex	Energy (kcal/mol)
LPxTG with MUC1	−635.4
LPxTG with MUC3A	−938.5
**LPxTG with MUC4**	**−1317.1**
**LPxTG with MUC12**	**−1122.9**
LPxTG with MUC13	−952.1

## Data Availability

Genome sequence is available in NCBI GenBank under the accession number JABFCW020000000, JABFCU020000000, NFZO02000000 and JABFCV020000000.

## Supplementary Materials

The following supporting information can be downloaded at: https://www.mdpi.com/article/10.3390/microorganisms12091771/s1, Figure S1: Gram-stained images of exclusion of *S.* Typhimurium from HT-29 cell line by *S. clausii* strains. Table S1: Genes related to adhesion to intestinal epithelium detected in *S. clausii* strains.

## References

[1] Hill C., Guarner F., Reid G., Gibson G.R., Merenstein D.J., Pot B., Morelli L., Canani R.B., Flint H.J., Salminen S. (2014). The International Scientific Association for Probiotics and Prebiotics consensus statement on the scope and appropriate use of the term probiotic. Nat. Rev. Gastroenterol. Hepatol..

[2] Binda S., Hill C., Johansen E., Obis D., Pot B., Sanders M.E., Tremblay A., Ouwehand A.C. (2020). Criteria to qualify microorganisms as “probiotic” in foods and dietary supplements. Front. Microbiol..

[3] Fijan S., Frauwallner A., Langerholc T., Krebs B., ter Haar J.A., Heschl A., Mičetić Turk D., Rogelj I. (2019). Efficacy of using probiotics with antagonistic activity against pathogens of wound infections: An integrative review of literature. Biomed. Res. Int..

[4] Anselmo A.C., McHugh K.J., Webster J., Langer R., Jaklenec A. (2016). Layer by layer encapsulation of probiotics for delivery to the microbiome. Adv. Mater..

[5] Bernardeau M., Lehtinen M.J., Forssten S.D., Nurminen P. (2017). Importance of the gastrointestinal life cycle of *Bacillus* for probiotic functionality. J. Food Sci. Technol..

[6] Łubkowska B., Jeżewska-Frąckowiak J., Sroczyński M., Dzitkowska-Zabielska M., Bojarczuk A., Skowron P.M., Cięszczyk P. (2023). Analysis of industrial *Bacillus* species as potential probiotics for dietary supplements. Microorganisms.

[7] Joshi A., Thite S., Karodi P., Joseph N., Lodha T. (2022). Corrigendum: *Alkalihalobacterium elongatum* gen. nov. sp. nov.: An antibiotic-producing bacterium isolated from Lonar Lake and reclassification of the genus *Alkalihalobacillus* into seven novel genera. Front. Microbiol..

[8] Ghelardi E., Mazzantini D., Celandroni F., Calvigioni M., Panattoni A., Lupetti A., de Fer B.B., Perez M. (2023). Analysis of the microbial content of probiotic products commercialized worldwide and survivability in conditions mimicking the human gut environment. Front. Microbiol..

[9] Nista E.C., Candelli M., Cremonini F., Cazzato I.A., Zocco M.A., Franceschi F., Cammarota G., Gasbarrini G., Gasbarrini A. (2004). *Bacillus clausii* therapy to reduce side-effects of anti-*Helicobacter pylori* treatment: Randomized, double-blind, placebo controlled trial. Aliment. Pharmacol. Ther..

[10] Acosta-Rodríguez-Bueno C.P., Abreu y Abreu A.T., Guarner F., Guno M.J.V., Pehlivanoğlu E., Perez M. (2022). *Bacillus clausii* for gastrointestinal disorders: A narrative literature review. Adv. Ther..

[11] Ianiro G., Rizzatti G., Plomer M., Lopetuso L., Scaldaferri F., Franceschi F., Cammarota G., Gasbarrini A. (2018). *Bacillus clausii* for the treatment of acute diarrhea in children: A systematic review and meta-analysis of randomized controlled trials. Nutrients.

[12] Khokhlova E., Colom J., Simon A., Mazhar S., García-Lainez G., Llopis S., Gonzalez N., Enrique-López M., Álvarez B., Martorell P. (2023). Immunomodulatory and antioxidant properties of a novel potential probiotic *Bacillus clausii* CSI08. Microorganisms.

[13] Tuomola E.M., Salminen S.J. (1998). Adhesion of some probiotic and dairy *Lactobacillus* strains to Caco-2 cell cultures. Int. J. Food Microbiol..

[14] Ouwehand A.C., Tuomola E.M., Tölkkö S., Salminen S. (2001). Assessment of adhesion properties of novel probiotic strains to human intestinal mucus. Int. J. Food Microbiol..

[15] Štyriak I., Nemcova R., Chang Y.H., Ljungh Å. (2003). Binding of extracellular matrix molecules by probiotic bacteria. Lett. Appl. Microbiol..

[16] Ahire J.J., Kashikar M.S., Madempudi R.S. (2021). Comparative accounts of probiotic properties of spore and vegetative cells of *Bacillus clausii* UBBC07 and in silico analysis of probiotic function. 3 Biotech.

[17] Jeon H.L., Lee N.K., Yang S.J., Kim W.S., Paik H.D. (2017). Probiotic characterization of *Bacillus subtilis* P223 isolated from kimchi. Food Sci. Biotechnol..

[18] Mazzantini D., Calvigioni M., Celandroni F., Lupetti A., Ghelardi E. (2022). In vitro assessment of probiotic attributes for strains contained in commercial formulations. Sci. Rep..

[19] Bubnov R.V., Babenko L.P., Lazarenko L.M., Mokrozub V.V., Spivak M.Y. (2018). Specific properties of probiotic strains: Relevance and benefits for the host. EPMA J..

[20] Vecchione A., Celandroni F., Mazzantini D., Senesi S., Lupetti A., Ghelardi E. (2018). Compositional quality and potential gastrointestinal behavior of probiotic products commercialized in Italy. Front. Med..

[21] Jacobsen C.N., Rosenfeldt Nielsen V., Hayford A.E., Møller P.L., Michaelsen K.F., Paerregaard A., Sandstrom B., Tvede M., Jakobsen M. (1999). Screening of probiotic activities of forty-seven strains of *Lactobacillus* spp. by in vitro techniques and evaluation of the colonization ability of five selected strains in humans. Appl. Environ. Microbiol..

[22] Lim Y.H., Foo H.L., Loh T.C., Mohamad R., Abdullah N. (2019). Comparative studies of versatile extracellular proteolytic activities of lactic acid bacteria and their potential for extracellular amino acid productions as feed supplements. J. Anim. Sci. Biotechnol..

[23] Vijayakumari Nadaraja A., Jayakumaran Nair A., Prameela M., Hari N., Balakrishnan N. (2018). Evaluation of currently employed food preservation conditions to tackle biofilm forming food pathogens. J. Food Saf..

[24] Mu G., Gao Y., Tuo Y., Li H., Zhang Y., Qian F., Jiang S. (2018). Assessing and comparing antioxidant activities of lactobacilli strains by using different chemical and cellular antioxidant methods. J. Dairy Sci..

[25] Tarrah A., dos Santos Cruz B.C., Sousa Dias R., da Silva Duarte V., Pakroo S., de Oliveira L.L., Gouveia Peluzio M.C., Corich V., Giacomini A., Oliveira de Paula S. (2021). *Lactobacillus paracasei* DTA81, a cholesterol-lowering strain having immunomodulatory activity, reveals gut microbiota regulation capability in BALB/c mice receiving high-fat diet. J. Appl. Microbiol..

[26] Mıdık F., Tokatlı M., Bağder Elmacı S., Özçelik F. (2020). Influence of different culture conditions on exopolysaccharide production by indigenous lactic acid bacteria isolated from pickles. Arch. Microbiol..

[27] Sharma S., Kanwar S.S. (2017). Adherence potential of indigenous lactic acid bacterial isolates obtained from fermented foods of Western Himalayas to intestinal epithelial Caco-2 and HT-29 cell lines. J. Food Sci. Technol..

[28] Lebeer S., Claes I., Tytgat H.L., Verhoeven T.L., Marien E., von Ossowski I., Reunanen J., Palva A., de Vos W.M., de Keersmaecker S.C. (2012). Functional analysis of *Lactobacillus rhamnosus* GG pili in relation to adhesion and immunomodulatory interactions with intestinal epithelial cells. Appl. Environ. Microbiol..

[29] Inturri R., Stivala A., Sinatra F., Morrone R., Blandino G. (2014). Scanning electron microscopy observation of adhesion properties of *Bifidobacterium longum* W11 and chromatographic analysis of its exopolysaccaride. Food Nutr. Sci..

[30] Choi A.R., Patra J.K., Kim W.J., Kang S.S. (2018). Antagonistic activities and probiotic potential of lactic acid bacteria derived from a plant-based fermented food. Front. Microbiol..

[31] Muñoz-Provencio D., Llopis M., Antolín M., De Torres I., Guarner F., Pérez-Martínez G., Monedero V. (2009). Adhesion properties of *Lactobacillus casei* strains to resected intestinal fragments and components of the extracellular matrix. Arch. Microbiol..

[32] Rokana N., Singh B.P., Thakur N., Sharma C., Gulhane R.D., Panwar H. (2018). Screening of cell surface properties of potential probiotic lactobacilli isolated from human milk. J. Dairy Res..

[33] Han Q., Kong B., Chen Q., Sun F., Zhang H. (2017). In vitro comparison of probiotic properties of lactic acid bacteria isolated from Harbin dry sausages and selected probiotics. J. Funct. Foods.

[34] Collado M.C., Isolauri E., Salminen S. (2008). Specific probiotic strains and their combinations counteract adhesion of *Enterobacter sakazakii* to intestinal mucus. FEMS Microbiol. Lett..

[35] Aziz R.K., Bartels D., Best A.A., DeJongh M., Disz T., Edwards R.A., Formsma K., Gerdes S., Glass E.M., Kubal M. (2008). The RAST Server: Rapid annotations using subsystems technology. BMC Genom..

[36] Carattoli A., Zankari E., García-Fernández A., Voldby Larsen M., Lund O., Villa L., Møller Aarestrup F., Hasman H. (2014). In silico detection and typing of plasmids using PlasmidFinder and plasmid multilocus sequence typing. Antimicrob. Agents Chemother..

[37] Malberg Tetzschner A.M., Johnson J.R., Johnston B.D., Lund O., Scheutz F. (2020). In silico genotyping of *Escherichia coli* isolates for extraintestinal virulence genes by use of whole-genome sequencing data. J. Clin. Microbiol..

[38] Van Heel A.J., de Jong A., Song C., Viel J.H., Kok J., Kuipers O.P. (2018). BAGEL4: A user-friendly web server to thoroughly mine RiPPs and bacteriocins. Nucleic Acids Res..

[39] Yang J., Zhang Y. (2015). I-TASSER server: New development for protein structure and function predictions. Nucleic Acids Res..

[40] Pieper U., Webb B.M., Barkan D.T., Schneidman-Duhovny D., Schlessinger A., Braberg H., Yang Z., Meng E.C., Pettersen E.F., Huang C.C. (2010). ModBase, a database of annotated comparative protein structure models, and associated resources. Nucleic Acids Res..

[41] Kozakov D., Hall D.R., Xia B., Porter K.A., Padhorny D., Yueh C., Beglov D., Vajda S. (2017). The ClusPro web server for protein–protein docking. Nat. Protoc..

[42] McFarland L.V., Evans C.T., Goldstein E.J. (2018). Strain-specificity and disease-specificity of probiotic efficacy: A systematic review and meta-analysis. Front. Med..

[43] Malki A.A., Yoon S.H., Firoz A., Ali H.M., Park Y.H., Hor Y.Y., Rather I.A. (2022). Characterization of new probiotic isolates from fermented ajwa dates of madinah and their anti-inflammatory potential. Appl. Sci..

[44] Aleman R.S., Yadav A. (2023). Systematic Review of Probiotics and Their Potential for Developing Functional Nondairy Foods. Appl. Microbiol..

[45] Cenci G., Trotta F., Caldini G. (2006). Tolerance to challenges miming gastrointestinal transit by spores and vegetative cells of *Bacillus clausii*. J. Appl. Microbiol..

[46] Goderska K., Agudo Pena S., Alarcon T. (2018). *Helicobacter pylori* treatment: Antibiotics or probiotics. Appl. Microbiol. Biotechnol..

[47] Abbrescia A., Palese L.L., Papa S., Gaballo A., Alifano P., Sardanelli A.M. (2014). Antibiotic sensitivity of *Bacillus clausii* strains in commercial preparation. Clin. Immunol. Endocr. Metab. Drugs.

[48] Lakshmi S.G., Jayanthi N., Saravanan M., Ratna M.S. (2017). Safety assesment of *Bacillus clausii* UBBC07, a spore forming probiotic. Toxicol. Rep..

[49] Khatri I., Sharma G., Subramanian S. (2019). Composite genome sequence of *Bacillus clausii*, a probiotic commercially available as Enterogermina^®^, and insights into its probiotic properties. BMC Microbiol..

[50] Urdaci M.C., Bressollier P., Pinchuk I. (2004). *Bacillus clausii* probiotic strains: Antimicrobial and immunomodulatory activities. J. Clin. Gastroenterol..

[51] Sharma J., Upadhya S. (2015). Determination of antimicrobial potential of *Saccharomyces boulardii* and *Bacillus clausii* against some community acquired pathogens in vitro study. Int. J. Pharm. Sci. Res..

[52] Grove T.L., Himes P.M., Hwang S., Yumerefendi H., Bonanno J.B., Kuhlman B., Almo S.C., Bowers A.A. (2017). Structural insights into thioether bond formation in the biosynthesis of sactipeptides. J. Am. Chem. Soc..

[53] Cotter P.D., Ross R.P., Hill C. (2013). Bacteriocins—A viable alternative to antibiotics?. Nat. Rev. Microbiol..

[54] Rahmdel S., Shekarforoush S.S., Hosseinzadeh S., Torriani S., Gatto V. (2019). Antimicrobial spectrum activity of bacteriocinogenic *Staphylococcus* strains isolated from goat and sheep milk. J. Dairy Sci..

[55] Ahire J.J., Kashikar M.S., Madempudi R.S. (2020). Survival and germination of *Bacillus clausii* UBBC07 spores in in vitro human gastrointestinal tract simulation model and evaluation of clausin production. Front. Microbiol..

[56] Rochín-Medina J.J., Ramírez-Medina H.K., Rangel-Peraza J.G., Pineda-Hidalgo K.V., Iribe-Arellano P. (2018). Use of whey as a culture medium for *Bacillus clausii* for the production of protein hydrolysates with antimicrobial and antioxidant activity. Food Sci. Technol. Int..

[57] Ripert G., Racedo S.M., Elie A.M., Jacquot C., Bressollier P., Urdaci M.C. (2016). Secreted compounds of the probiotic *Bacillus clausii* strain O/C inhibit the cytotoxic effects induced by *Clostridium difficile* and *Bacillus cereus* toxins. Antimicrob. Agents Chemother..

[58] Patel C., Patel P., Acharya S. (2020). Therapeutic prospective of a spore-forming probiotic---Bacillus clausii UBBC07 against acetaminophen-induced uremia in rats. Probiotics Antimicrob. Proteins.

[59] Paparo L., Tripodi L., Luccioni C., Bruno C., Pisapia L., Damiano C., Pastore L., Berni Canani R. (2020). Protective action of *Bacillus clausii* probiotic strains in an in vitro model of Rotavirus infection.. Sci. Rep..

[60] Ooi L.G., Liong M.T. (2010). Cholesterol-lowering effects of probiotics and prebiotics: A review of in vivo and in vitro findings. Int. J. Mol. Sci..

[61] Gorreja F., Walker W.A. (2022). The potential role of adherence factors in probiotic function in the gastrointestinal tract of adults and pediatrics: A narrative review of experimental and human studies. Gut Microbes.

[62] Lau L.Y.J., Quek S.Y. (2024). Probiotics: Health benefits, food application, and colonization in the human gastrointestinal tract. Food Bioeng..

[63] Monteagudo-Mera A., Rastall R.A., Gibson G.R., Charalampopoulos D., Chatzifragkou A. (2019). Adhesion mechanisms mediated by probiotics and prebiotics and their potential impact on human health. Appl. Microbiol. Biotechnol..

[64] Zawistowska-Rojek A., Kośmider A., Stępień K., Tyski S. (2022). Adhesion and aggregation properties of *Lactobacillaceae* strains as protection ways against enteropathogenic bacteria. Arch. Microbiol..

[65] Lea T. (2015). Caco-2 cell line. The Impact of Food Bioactives on Health: In Vitro and Ex Vivo Models.

[66] Haeri A., Khodaii Z., Ghaderian S.M.H., Tabatabaei Panah A.S., Akbarzadeh Najar R. (2012). Comparison of adherence patterns of a selection of probiotic bacteria to Caco-2, HEp-2, and T84 cell lines. Ann. Microbiol..

[67] Sambuy Y., De Angelis I., Ranaldi G., Scarino M.L., Stammati A., Zucco F. (2005). The Caco-2 cell line as a model of the intestinal barrier: Influence of cell and culture-related factors on Caco-2 cell functional characteristics. Cell Biol. Toxicol..

[68] Kleiveland C.R. (2015). Co-cultivation of Caco-2 and HT-29MTX. The Impact of Food Bioactives on Health: In Vitro and Ex Vivo Models.

[69] De Vecchi E., Nicola L., Zanini S., Drago L. (2008). In vitro screening of probiotic characteristics of some Italian products. J. Chemother..

[70] Haddaji N., Mahdhi A.K., Krifi B., Ismail M.B., Bakhrouf A. (2015). Change in cell surface properties of *Lactobacillus casei* under heat shock treatment. FEMS Microbiol. Lett..

[71] Lee Y.K., Puong K.Y., Ouwehand A.C., Salminen S. (2003). Displacement of bacterial pathogens from mucus and Caco-2 cell surface by lactobacilli. J. Appl. Microbiol..

[72] Vélez M.P., Hermans K., Verhoeven T.L.A., Lebeer S.E., Vanderleyden J., De Keersmaecker S.C.J. (2007). Identification and characterization of starter lactic acid bacteria and probiotics from Columbian dairy products. J. Appl. Microbiol..

[73] Andriantsoanirina V., Teolis A.C., Xin L.X., Butel M.J., Aires J. (2014). *Bifidobacterium longum* and *Bifidobacterium breve* isolates from preterm and full term neonates: Comparison of cell surface properties. Anaerobe.

[74] Krausova G., Hyrslova I., Hynstova I. (2019). In vitro evaluation of adhesion capacity, hydrophobicity, and auto-aggregation of newly isolated potential probiotic strains. Fermentation.

[75] Ehrmann M.A., Kurzak P., Bauer J., Vogel R.F. (2002). Characterization of lactobacilli towards their use as probiotic adjuncts in poultry. J. Appl. Microbiol..

[76] Vinderola C.G., Medici M., Perdigon G. (2004). Relationship between interaction sites in the gut, hydrophobicity, mucosal immunomodulating capacities and cell wall protein profiles in indigenous and exogenous bacteria. J. Appl. Microbiol..

[77] García-Cayuela T., Korany A.M., Bustos I., de Cadiñanos L.P.G., Requena T., Peláez C., Martínez-Cuesta M.C. (2014). Adhesion abilities of dairy *Lactobacillus plantarum* strains showing an aggregation phenotype. Food Res. Int..

[78] Campana R., van Hemert S., Baffone W. (2017). Strain-specific probiotic properties of lactic acid bacteria and their interference with human intestinal pathogens invasion. Gut Pathog..

[79] Chaffanel F., Charron-Bourgoin F., Soligot C., Kebouchi M., Bertin S., Payot S., Le Roux Y., Leblond-Bourget N. (2018). Surface proteins involved in the adhesion of *Streptococcus salivarius* to human intestinal epithelial cells. Appl. Microbiol. Biotechnol..

[80] Kapczynski D.R., Meinersmann R.J., Lee M.D. (2000). Adherence of *Lactobacillus* to intestinal 407 cells in culture correlates with fibronectin binding. Curr. Microbiol..

[81] Claesson M.J., Li Y., Leahy S., Canchaya C., van Pijkeren J.P., Cerdeño-Tárraga A.M., Parkhill J., Flynn S., O’Sullivan G.C., Collins J.K. (2006). Multireplicon genome architecture of *Lactobacillus salivarius*. Proc. Natl. Acad. Sci. USA.

[82] Kebouchi M., Galia W., Genay M., Soligot C., Lecomte X., Awussi A.A., Perrin C., Roux E., Dary-Mourot A., Le Roux Y. (2016). Implication of sortase-dependent proteins of *Streptococcus thermophilus* in adhesion to human intestinal epithelial cell lines and bile salt tolerance. Appl. Microbiol. Biotechnol..

[83] Kainulainen V., Korhonen T.K. (2014). Dancing to another tune—Adhesive moonlighting proteins in bacteria. Biology.

[84] Nagarajan R., Sankar S., Ponnuraj K. (2019). Crystal structure of GAPDH of *Streptococcus agalactiae* and characterization of its interaction with extracellular matrix molecules. Microb. Pathog..

[85] Nelson D., Goldstein J.M., Boatright K., Harty D.W.S., Cook S.L., Hickman P.J., Potempa J., Travis J., Mayo J.A. (2001). pH-regulated secretion of a glyceraldehyde-3-phosphate dehydrogenase from *Streptococcus gordonii* FSS2: Purification, characterization, and cloning of the gene encoding this enzyme. J. Dent. Res..

[86] Kinoshita H., Uchida H., Kawai Y., Kawasaki T., Wakahara N., Matsuo H., Watanabe M., Kitazawa H., Ohnuma S., Miura K. (2008). Cell surface *Lactobacillus plantarum* LA 318 glyceraldehyde-3-phosphate dehydrogenase (GAPDH) adheres to human colonic mucin. J. Appl. Microbiol..

[87] Antikainen J., Kuparinen V., Lähteenmäki K., Korhonen T.K. (2007). Enolases from Gram-positive bacterial pathogens and commensal lactobacilli share functional similarity in virulence-associated traits. FEMS Immunol. Med. Microbiol..

[88] Sanchez B., Bressollier P., Urdaci M.C. (2008). Exported proteins in probiotic bacteria: Adhesion to intestinal surfaces, host immunomodulation and molecular cross-talking with the host. FEMS Immunol. Med. Microbiol..

[89] Granato D., Bergonzelli G.E., Pridmore R.D., Marvin L., Rouvet M., Corthésy-Theulaz I.E. (2004). Cell surface-associated elongation factor Tu mediates the attachment of *Lactobacillus johnsonii* NCC533 (La1) to human intestinal cells and mucins. Infect. Immun..

[90] Bergonzelli G.E., Granato D., Pridmore R.D., Marvin-Guy L.F., Donnicola D., Corthésy-Theulaz I.E. (2006). GroEL of *Lactobacillus johnsonii* La1 (NCC 533) is cell surface associated: Potential role in interactions with the host and the gastric pathogen *Helicobacter pylori*. Infect. Immun..

[91] Marraffini L.A., DeDent A.C., Schneewind O. (2006). Sortases and the art of anchoring proteins to the envelopes of gram-positive bacteria. Microbiol. Mol. Biol. Rev..

[92] Van Pijkeren J.P., Canchaya C., Ryan K.A., Li Y., Claesson M.J., Sheil B., Steidler L., O’Mahony L., Fitzgerald G.F., van Sinderen D. (2006). Comparative and functional analysis of sortase-dependent proteins in the predicted secretome of *Lactobacillus salivarius* UCC118. Appl. Environ. Microbiol..

[93] Kumar R., Bansal P., Singh J., Dhanda S., Bhardwaj J.K. (2020). Aggregation, adhesion and efficacy studies of probiotic candidate *Pediococcus acidilactici* NCDC 252: A strain of dairy origin. World J. Microbiol. Biotechnol..

[94] Das J.K., Mahapatra R.K., Patro S., Goswami C., Suar M. (2016). *Lactobacillus acidophilus* binds to MUC3 component of cultured intestinal epithelial cells with highest affinity. FEMS Microbiol. Lett..

