# Supplementary material for: Strain-Dependent Adhesion Variations of Shouchella clausii Isolated from Healthy Human Volunteers: A Study on Cell Surface Properties and Potential Probiotic Benefits
Source: Microorganisms. 2024 Aug 27;12(9):1771. doi: 10.3390/microorganisms12091771 (PMC11434523; doi:10.3390/microorganisms12091771)
Supplement: Supplementary file 1 [file microorganisms-12-01771-s001.zip › microorganisms-3140897-supplementary.pdf]

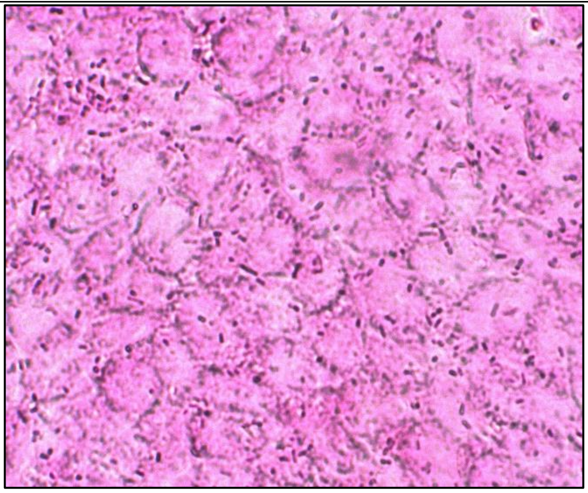

(A) Exclusion of *S.* Typhimurium by B619/R

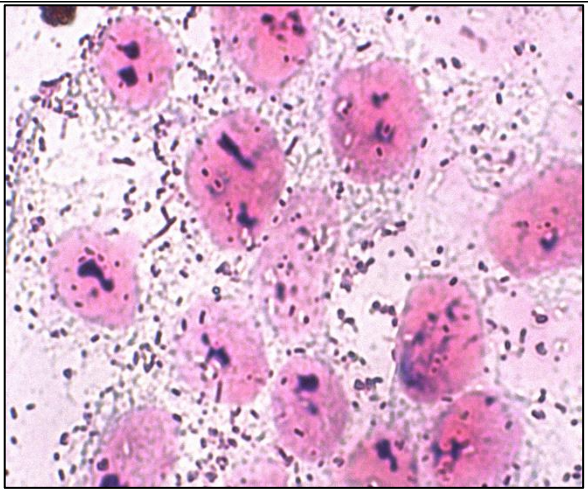

(B) Exclusion of *S.* Typhimurium by B603/Nb

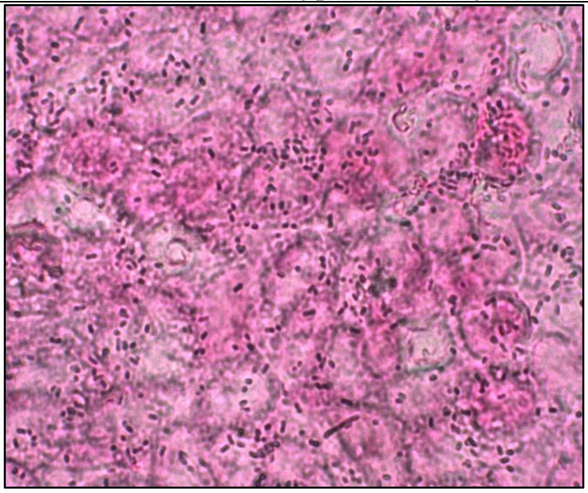

(C) Exclusion of *S.* Typhimurium by B106

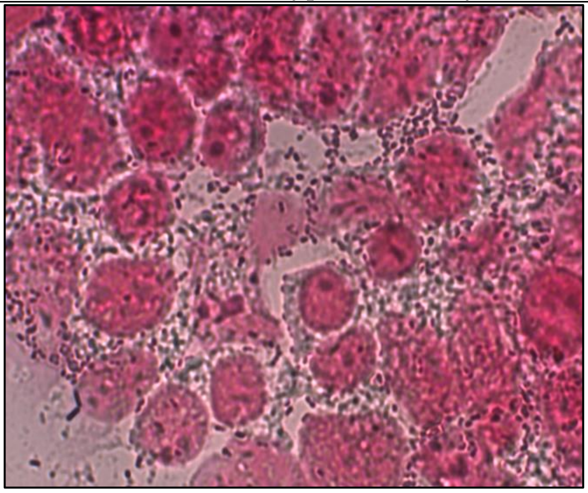

(D) Exclusion of *S.* Typhimurium by B637/Nm

Figure S1: Gram-stained images of exclusion of *S.* Typhimurium from HT-29 cell line by *S. clausii* strains

**Table S1: Genes related to adhesion to intestinal epithelium detected in *S. clausii* strains**

| Genes involved in colonization of intestinal mucosa | Genes detected in *S. clausii* strains | | | |
|---|---|---|---|---|
| | B619/R | B603/Nb | B106 | B637/Nm |
| Elongation factor Tu | fig\|6666666.1068537.peg.844 | fig\|6666666.1068538.peg.951 | fig\|6666666.1068539.peg.838 | fig\|6666666.1068540.peg.840 |
| Collagen adhesion protein | fig\|6666666.1068537.peg.1169 | fig\|6666666.1068538.peg.1219 | fig\|6666666.1068539.peg.1154 | fig\|6666666.1068540.peg.1163 |
| Fibronectin binding protein | fig\|6666666.1068537.peg.3106 | fig\|6666666.1068538.peg.2627 | fig\|6666666.1068539.peg.2728 | fig\|6666666.1068540.peg.3132 |
| Enolase | fig\|6666666.1068537.peg.4396 | fig\|6666666.1068538.peg.4379 | fig\|6666666.1068539.peg.4410 | fig\|6666666.1068540.peg.4418 |
| Cell wall surface anchor family protein (LPxTG motif) | fig\|6666666.1068537.peg.456<br>fig\|6666666.1068537.peg.1870<br>fig\|6666666.1068537.peg.1877 | fig\|6666666.1068538.peg.21<br>fig\|6666666.1068538.peg.2419 | fig\|6666666.1068539.peg.180<br>fig\|6666666.1068539.peg.2526<br>fig\|6666666.1068539.peg.2908 | x |
| Sortase A | fig\|6666666.1068537.peg.1170 | fig\|6666666.1068538.peg.1220 | fig\|6666666.1068539.peg.1155 | fig\|6666666.1068540.peg.1164 |
| LspA (Lipoprotein signal peptidase) | fig\|6666666.1068537.peg.3091 | fig\|6666666.1068538.peg.2612 | fig\|6666666.1068539.peg.2713 | fig\|6666666.1068540.peg.3117 |
| EPS biosynthesis gene cluster | | | | |

| | | | | |
|---|---|---|---|---|
| Manganese-dependent protein-tyrosine phosphatase | fig\|6666666.1068537.peg.4129 | fig\|6666666.1068538.peg.2816 | fig\|6666666.1068539.peg.4146 | fig\|6666666.1068540.peg.4152 |
| Tyrosine-protein kinase EpsD | fig\|6666666.1068537.peg.4130 | fig\|6666666.1068538.peg.2817 | fig\|6666666.1068539.peg.4147 | fig\|6666666.1068540.peg.4153 |
| Tyrosine-protein kinase transmembrane modulator EpsC | fig\|6666666.1068537.peg.4131 | fig\|6666666.1068538.peg.2818 | fig\|6666666.1068539.peg.4148 | fig\|6666666.1068540.peg.4154 |
| Undecaprenyl-phosphate galactosephosphotransferase | fig\|6666666.1068537.peg.428 | fig\|6666666.1068538.peg.541 | fig\|6666666.1068539.peg.404 | fig\|6666666.1068540.peg.282 |
| Flagellar motor switch protein FliG, FliM, FliN | fig\|6666666.1068537.peg.3164 fig\|6666666.1068537.peg.3174 fig\|6666666.1068537.peg.3175 | fig\|6666666.1068538.peg.2686 fig\|6666666.1068538.peg.2696 fig\|6666666.1068538.peg.2697 | fig\|6666666.1068539.peg.2787 fig\|6666666.1068539.peg.2797 fig\|6666666.1068539.peg.2798 | fig\|6666666.1068540.peg.3190 fig\|6666666.1068540.peg.3200 fig\|6666666.1068540.peg.3201 |
| Flagellar hook-associated protein FlgK | fig\|6666666.1068537.peg.4329 | fig\|6666666.1068538.peg.4313 | fig\|6666666.1068539.peg.4343 | fig\|6666666.1068540.peg.4350 |
| Flagellar hook-associated protein FlgL | fig\|6666666.1068537.peg.4330 | fig\|6666666.1068538.peg.4314 | fig\|6666666.1068539.peg.4344 | fig\|6666666.1068540.peg.4351 |

| | | | | |
|---|---|---|---|---|
| Flagellar hook-associated protein FliD | fig\|6666666.1068537.peg.4334 | fig\|6666666.1068538.peg.4318 | fig\|6666666.1068539.peg.4348 | fig\|6666666.1068540.peg.4355 |
| Flagellar biosynthesis proteins FlhA, FlhB, FliP, FliQ, FliR, FliS | fig\|6666666.1068537.peg.3182<br>fig\|6666666.1068537.peg.3181<br>fig\|6666666.1068537.peg.3178<br>fig\|6666666.1068537.peg.3179<br>fig\|6666666.1068537.peg.3180<br>fig\|6666666.1068537.peg.4335 | fig\|6666666.1068538.peg.2704<br>fig\|6666666.1068538.peg.2703<br>fig\|6666666.1068538.peg.2700<br>fig\|6666666.1068538.peg.2701<br>fig\|6666666.1068538.peg.2702<br>fig\|6666666.1068538.peg.4319 | fig\|6666666.1068539.peg.2805<br>fig\|6666666.1068539.peg.2804<br>fig\|6666666.1068539.peg.2801<br>fig\|6666666.1068539.peg.2802<br>fig\|6666666.1068539.peg.2803<br>fig\|6666666.1068539.peg.4349 | fig\|6666666.1068540.peg.3208<br>fig\|6666666.1068540.peg.3207<br>fig\|6666666.1068540.peg.3204<br>fig\|6666666.1068540.peg.3205<br>fig\|6666666.1068540.peg.3206<br>fig\|6666666.1068540.peg.4356 |
| Triosephosphate isomerase | fig\|6666666.1068537.peg.4394 | fig\|6666666.1068538.peg.4377 | fig\|6666666.1068539.peg.4408 | fig\|6666666.1068540.peg.4416 |
